# Molecular Wires for Efficient Long-Distance Triplet Energy Transfer

**DOI:** 10.1021/acs.jpclett.2c02616

**Published:** 2022-10-10

**Authors:** Spyroulla A. Mavrommati, Spiros S. Skourtis

**Affiliations:** Department of Physics, University of Cyprus, P.O. Box 20537, Nicosia 1678, Cyprus

## Abstract

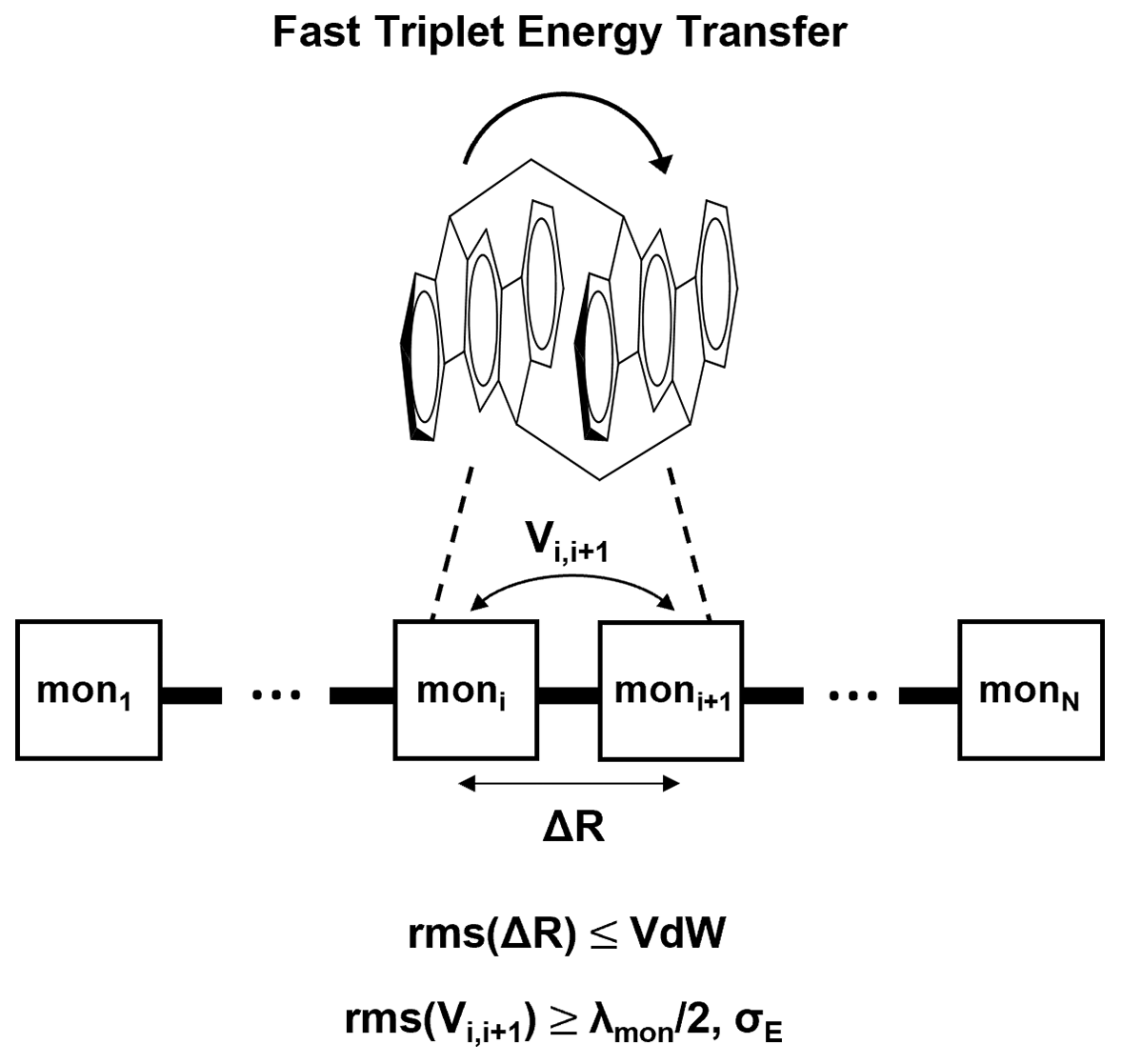

We propose design rules for building organic molecular bridges that enable coherent long-distance triplet-exciton transfer. Using these rules, we describe example polychromophoric structures with low inner-sphere exciton reorganization energies, low static and dynamic disorder, and enhanced π-stacking interactions between nearest-neighbor chromophores. These features lead to triplet-exciton eigenstates that are delocalized over several units at room temperature. The use of such bridges in donor–bridge–acceptor assemblies enables fast triplet-exciton transport over very long distances that is rate-limited by the donor–bridge injection and bridge–acceptor trapping rates.

Triplet-exciton transfer (TET) is an important process in photosynthetic photoprotection and is central to the harvesting of triplet excitons (TE’s) in a variety of contexts such as catalysis, photovoltaics, and energy conversion [e.g., singlet fission (SF) and triplet–triplet annihilation].^1−9^ For many applications, it is desirable to achieve fast, directed, and long-distance TET. For example, in SF reactions that produce a correlated TE pair, it is important to separate the two TE products via fast TET to distinct locations, to avoid the back-reaction.^10−14^

Implementing directed long-distance ΤΕΤ on a single-molecule scale requires building molecular linkers (bridges) that connect TE donors (D) to acceptors (A).^2,15−23^ It is known that the speed of bridge-mediated D-to-A singlet-exciton transfer (SET) may be improved by enhanced π-stacking interactions between nearest-neighbor molecular bridge (B) units linking D and A.^24,25^ The π-stacking amplifies the nearest-neighbor SET couplings (*V*^SET^), leading to delocalized bridge singlet excitons (SE’s) that channel D-to-A SET.^26−32^ There are many examples of molecular assemblies with enhanced π-stacking interactions.^33−38^

However, a bridge with a large *V*^SET^ that supports fast SET will not support equally fast TET because the TET coupling between adjacent bridging units (*V*^TET^) is generally much weaker than *V*^SET^ for interunit distances (Δ*R*) that are greater than van der Waals (VdW) (*V*^TET^ decays approximately as e^–βΔ*R*^, whereas *V*^SET^ decays as 1/Δ*R*^3^).^27,39−41^ To illustrate this point, we computed both *V*^SET^ and *V*^TET^ for some of the π-stacked systems mentioned above^34,42−44^ (see section 6 of the Supporting Information). Even for geometries with π-stacking distances slightly greater than VdW and with *V*^SET^ ≈ 0.1 eV, *V*^TET^ ≈ 0.01 eV (see Figure S8).

This known behavior explains why long-distance TET is an incoherent hopping process, characterized by slow and short hopping steps, in contrast to SET that may involve quasi-coherent or fully coherent transfer mechanisms with faster and longer steps (especially for π-stacked assemblies). Although the lifetimes of TE’s are much longer than those of SE’s, the slow speed of TET constrains the transport distance that can be achieved within these lifetimes. Thus, an improvement in the speed of TET in organic bridges, in conjunction with the long TE lifetimes, could greatly enhance the maximum distance of TET. Our goal is to show how to modify existing organic π-stacked bridges to transform them to molecular wires that enable fast and coherent TET over long distances. Given the importance of purely organic electronic devices, we focus on organic (rather than metal–organic) molecules. In addition, we consider bridge architectures with a π-stacking core that support excitonic states that are largely localized within the core. This characteristic makes it easier to protect the excitons from solvent and environmental effects.

We suggest that a molecular bridge that could support delocalized TE’s and enable coherent TET should be designed to (i) be homopolymeric, (ii) have very tight π-stacking between units (maximum interunit distance should be VdW), (iii) maintain the tight π-stacking in the presence of room-temperature disorder, and (iv) have low inner-sphere reorganization energy for TE formation within each monomer unit (the outer-sphere reorganization energy depends on the solvent and should also be minimized by use of nonpolar solvents). Similar design principles have been discussed in the context of SET in molecular nanocrystals, e.g., refs (28) and (32), using results of theoretical studies of transport efficiency (for electrons, holes, or excitons) based on tight-binding (multisite) models.^26−28,31,39,41,45,46^ The important parameters for transport in a model with identical sites and independently fluctuating site energies are the root-mean-square coupling (*V*_rms_) between nearest-neighbor sites (localized electronic states), the site reorganization energy λ, and the standard deviation of site energy *σ*_E_ induced by dynamic disorder. Several studies show that delocalized eigenstates and coherent or quasi-coherent transport are possible when *V*_rms_ ≥ *σ*_E_, λ/2 (e.g., see refs (28), (30−32), and (45)). This condition is relevant to identical nearest-neighbor sites *i, i* + 1 and is derived from the assumption that each site has independent energy fluctuations. The eigenstates of the multisite system will not localize on each of the sites if the nearest-neighbor coupling satisfies *V*_*i,i*__+1_ ≥ *U*^act^, where *U*^act^ = (*λ*_*i*_ + λ_*i*+1_)/4 = λ/2 (because *λ*_*i*_ = *λ*_*i*__+1_ = λ). Therefore, *V*_rms_ ≥ λ/2 is an approximate condition that needs to be satisfied to allow for the possibility of delocalized TE eigenstates. It does not always guarantee the existence of delocalized eigenstates; i.e., it is a necessary but not a sufficient condition for localization, because the total reorganization energy in *U*^act^ may be greater than the sum of the inner-sphere monomer contributions due to collective molecular and solvent motions. *V*_rms_ ≥ *σ*_E_ is also an approximate condition that characterizes coherent transport (), in addition to *σ*_V_ < *V*_ave_. We use the criterion *V*_rms_ ≥ *σ*_E_, λ/2 to screen for molecular architectures that may support coherent long-distance TET. The criterion is combined with electronic structure and molecular dynamic (MD) computations and with a model for coherent transport. For each structure, we verify that *σ*_V_ < *V*_ave_.

Consider a polymeric wire with identical monomer chromophores and identify the lowest exciton level of each monomer with a site level in a multisite system (the latter representing a homopolymer). Given that *V*^TET^ is generally weak, it follows that the primary goals in the design of a polychromophoric molecular wire for coherent TET are the minimization of the site (monomer) reorganization energy λ (λ = *λ*^mon^) and of *σ*_*E*_ (*σ*_*Ε*_ = σ_*Ε*_^mon^) and the maximization of *V*_rms_. Typical minimal values for inner-sphere λ in molecules are on the order of 0.1 eV,^24,29^ leading to a room-temperature *σ*_E_ ≈ 0.1 eV. Given the condition *V*_rms_ ≥ *σ*_E_, λ/2, the *V*_rms_ magnitudes should be at least 0.1 eV. Such magnitudes require at most VdW π-stacking distances that are not destroyed by conformational disorder. Below we explore some potential structures that could fulfill these parameter value requirements.

Vura-Weis and co-workers probed the dependence of the D-to-A TET mechanism on bridge length using benzophenone (Bp) as D, naphthalene (Nap) as A, and polyfluorene as B.^47^ The fluorene (F) monomers were connected via methylene linkers in face-to-face (approximately π-stacked) geometries, and the bridge length was varied from one to three F units (*Fn*, where *n* = 1–3) (Scheme 1A,B). The experiment involved transient triplet absorption measurements and showed that through-bridge tunneling mediates transport for the shortest bridge length (one F unit), while for larger bridge lengths, the transport mechanism is multistep thermally activated hopping. The TET times for the dimer and trimer bridges were 100–200 ps (minimum bridge lengths of 7–10.5 Å, respectively). In these systems, the deduced D-to-B injection times and B-to-A trapping times are similar.

**Scheme 1:**
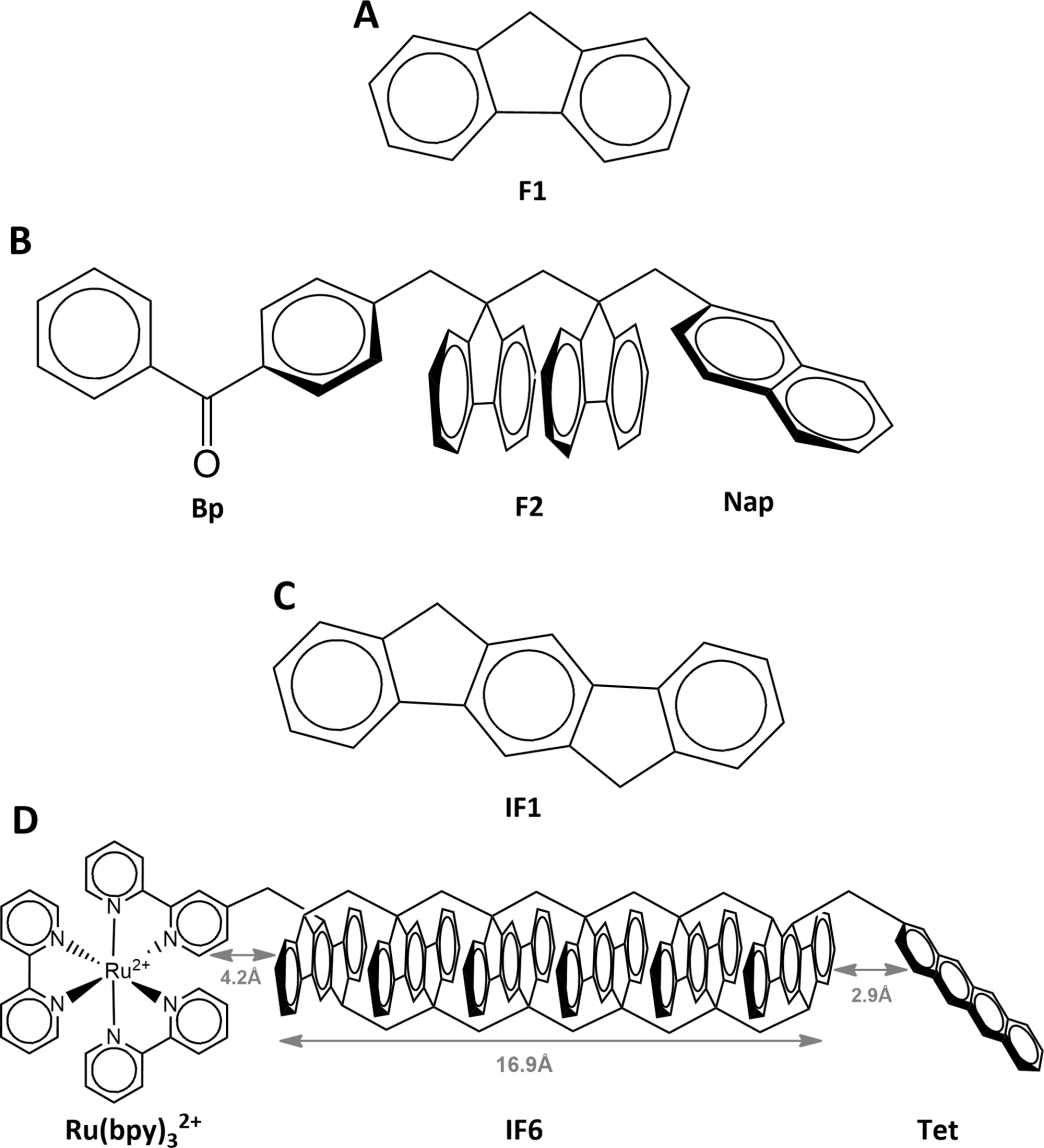
(A) Fluorene Monomer Bridging Unit (F1) Used in ref (47), (B) Structure of Bp–Fn–Nap Systems Used in ref (47), (C) Chemical Structure of the Proposed Indenofluorene (6,12-dihydroindeno[1,2-*b*]fluorene) Bridging Monomer (IF1), and (D) Molecular Structure of the Indenofluorene Hexamer (IF6) Bridge (16.9 Å length) The bridges contained one to three F units (F*n*, where *n* = 1–3). The monomers are constrained to a rigid π-stacked geometry through two methylene linkers, and the interchromophore distance is 2.8–3.0 Å. The IF6 bridge is linked to a Ru(bpy)_3_^2+^ D and a tetracene (Tet) A with distances of approximately 4.2 and 2.9 Å, respectively.

Electronic structure and MD simulations of the TET mechanism on the F*n* systems showed that the bridge TE’s are mostly localized in individual F units due to thermal disorder that involves twisting about the methylene linkers, destroying the F-to-F π-stacking interactions.^48^ In addition, the F-to-F *V*_rms_ was found to be small compared to the F–monomer TE reorganization energy. This result is consistent with the incoherent multistep hopping between F units for longer bridge lengths.

We can improve the F-based TE wires of ref (47) by transforming them into wires that fulfill the conditions mentioned above for coherent TET. Namely, one should minimize chromophore TE reorganization energy, enable tight π-stacking interactions between chromophores, and remedy the problem of conformational disorder. To this end, we propose polymers of indenofluorene (IF)-based monomer units brought to optimal π-stacked geometries by linking them with two methyl groups (Scheme 1C,D). Τhis linking brings the nearest-neighbor chromophores to an average distance of <3.4 Å and prevents torsional and slipping motions between the chromophores.

We tested the structural stability of the cofacial geometries and the π-stacking interactions in the dimer and the longer polymers (Scheme 1D) via room-temperature MD simulations. We also performed electronic structure computations to characterize the SE and TE spectra. The electronic structure methods included configuration interaction singles (CIS) and time-dependent density functional theory (TD-DFT) for the monomer and the larger systems, as well as higher-level *ab initio* approaches for the dimer (see the Supporting Information). The MD simulation results show that the tight π-stacking is maintained even at room temperature. Figure 1a shows examples of the TE eigenstates of the monomer (IF1), dimer (IF2), and decamer (IF10) computed with CIS for the minimum energy conformations. The energy difference between adjacent (in energy) TE eigenstates is high (e.g., 0.2 eV for IF10 to 0.3 eV for IF2), implying that the *V*^TET^ between neighboring units is large. The TE eigenstates are delocalized over several bridge units (see Figure 1b and the Supporting Information).

**Figure 1:**
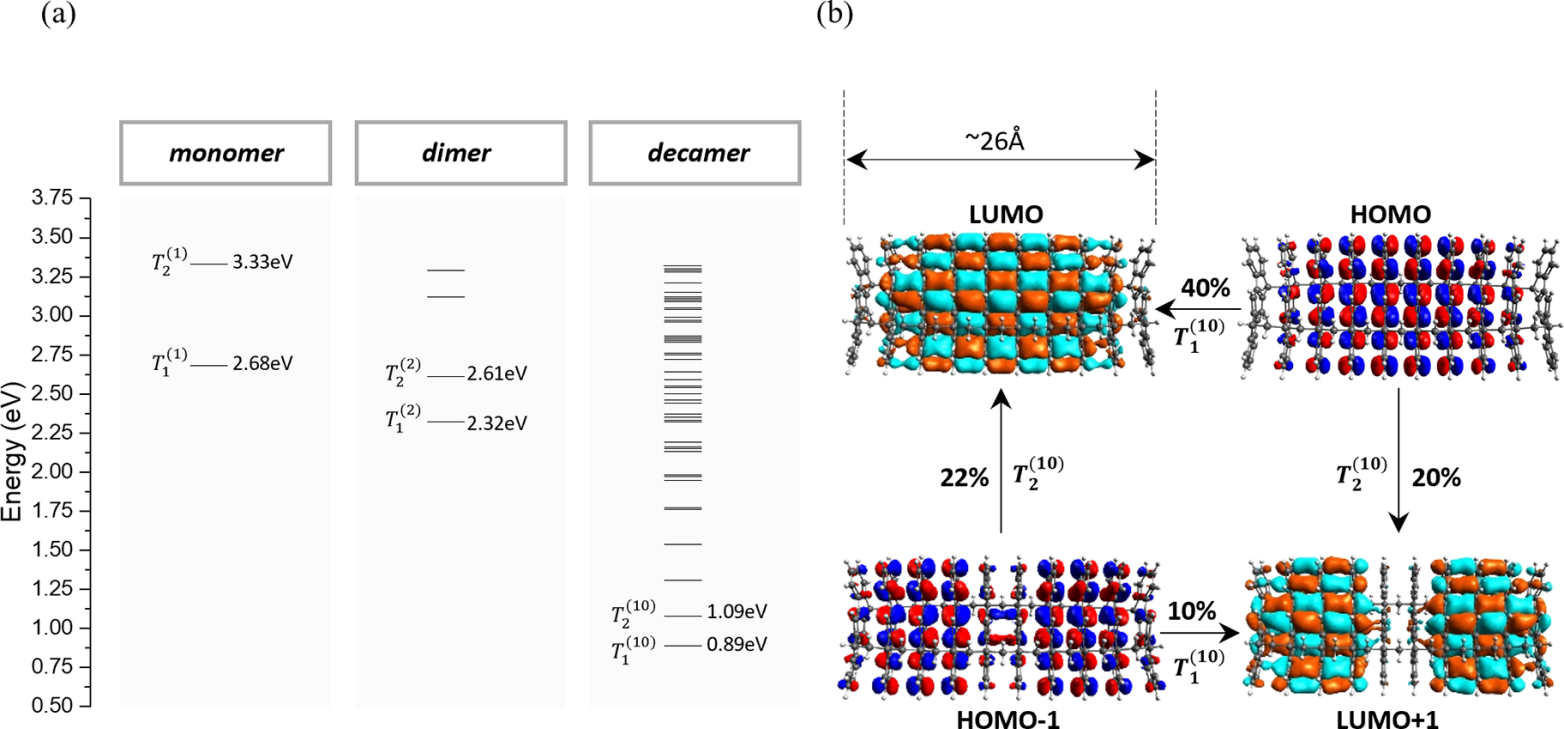
(a) TE states of the bridge type shown in Scheme 1 computed at the CIS/def2-SVP level of theory. Due to enhanced π-stacking, TE splittings are large [e.g.,  = 0.3 eV for the dimer]. For longer polymers, the TE band structure is stabilized. (b) Single-excitation molecular orbital contribution to the lowest two TE states of IF10 (computed with an isosurface value of 0.01). The figure shows only the largest contributions. The notations (1), (2), and (10) refer to monomer, dimer, and decamer bridges, respectively.

To explore the effects of thermal motion on the nearest-neighbor *V*^TET^, we first performed room-temperature MD simulations on the dimer. Using the MD trajectory structures, we computed (with CIS) the fluctuations in the energy splitting of the lowest two TE eigenstates, to estimate the nearest-neighbor coupling *V*^TET^ [Δ*Ε* = *Ε*_Τ_2_^(2)^_ – *E*_Τ_1_^(2)^_ ≈ 2*V*^TET^]. The MD-derived rms *V*^TET^ is large (*V*_rms_ = 0.13 eV) with a *V*_ave_ of 0.12 eV and a *σ*_V_ of 0.04 eV. These values indicate that the thermal structural distortions do not significantly reduce the monomer-to-monomer TET coupling.

To investigate the effects of thermal motion on the TE energies of the monomers, we computed, from the MD simulations on the dimer, the standard deviation (σ_*Ε*_^mon^) of the lowest TE energy *E* [*E* = *E*_Τ_1_^(1)^_] of each monomer fragment using CIS. For each MD dimer structure, we removed the methylene bridges that link the two IF units and capped the carbon atoms at positions 5′ and 11′ with hydrogens (see Figure S2). We found that σ_*Ε*_^mon^ = 0.12 eV; i.e., σ_*Ε*_^mon^ ≈ *V*_rms_. To test whether localized TE–polaron formation is likely in these polymeric systems, we compared *V*_rms_ to the activation energy for monomer-to-monomer TET. The activation energy is approximated by the equation *U*^act^ = (*λ*_D_ + *λ*_A_)/4, where *λ*_D_ and *λ*_A_ are the monomer reorganization energies (*λ*^mon^) as shown schematically in Figure S1. This notation implies that one monomer is the TE “donor” (D) and the other monomer the TE “acceptor” (A). We computed λ_D(A)_ = 0.27 eV (using TD-DFT and BP86^49^ functional) such that *U*^act^ = 0.14 eV, a value that is on the same order of magnitude as *V*_rms_. Because *V*_rms_ ≈ *U*^act^ = *λ*^mon^/2, we do not expect that TE-polaronic states are localized on a single monomer. In addition, as expected, .

To probe the delocalization of TE’s in the presence of disorder for longer polymers, we performed room-temperature MD simulations on the decamer followed by CIS computations on 3000 MD snapshots. From the snapshots, we computed averaged values of the HOMO and LUMO inverse participation ratios (IPR’s) (e.g., see refs (32), (50), and (51)). The HOMO and LUMO make large contributions to the lowest TE’s, e.g., Τ_1_^(10)^:40% HOMO → LUMO (for the other orbital contributions, see section 5 of the Supporting Information). As reference IPR values, we used those computed for the optimized geometry shown in Figure 1b. The computations show that the MD IPR values vary by approximately 27% with respect to the reference IPR. The results imply that thermal fluctuations largely preserve the delocalization of the TE’s shown in Figure 1b.

In summary, for these types of bridges, *V*_rms_ ≈ σ_E_^mon^ , *λ*^mon^/2. For this parameter regime, we estimate the intrabridge TET rate *k*_br_ (to be defined below) using an *N*-site tight-binding bridge Hamiltonian, *Ĥ* = ∑_*i*=1_^*N*^*E*|*i*⟩⟨*i*| + ∑_*i*=1_^*N*–1^(*V*|*i*⟩⟨*i* + 1| + *hc*) (see Figure 2a). Index *i* is the monomer number, and |*i*⟩ denotes the lowest TE eigenstate of the monomer (*E* is the TE energy, and *V* = *V*_rms_). We solve the Liouville equation for the density matrix
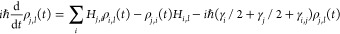
where the *γ*_*i*_ terms are monomer TE population-relaxation rates and the *γ*_*i, j*_ terms are pure dephasing rates for all *i*, *j* TE pairs.^52−56^ Each *γ*_*i*_ [*i* = 1 – (*N* – 1)] describes a phosphorescence decay rate with *γ*_*i*_ = *k*_B_^(ph)^ = (μs)^−1^ and *γ*_*N*_ = *k*_B_^(ph)^ + *k*_B→A_^(TET)^, where *k*_B→A_^(TET)^ is the TET rate from the *N*th monomer to an acceptor. The pure dephasing rates are set equal to , where *σ*_*i*(*j*)_ = σ_E_^mon^. This phenomenological model has been used in different contexts to study the transition from incoherent to coherent transport.^53−55,57^ It incorporates both diagonal dynamic disorder and population relaxation (we include approximately off-diagonal dynamic disorder by setting *V* = *V*_rms_). The model allows for analytical solutions of mean first passage times (MFPTs) as a function of its few parameters. These analytical solutions can be used to predict the approximate dependence of the intrabridge TET rate on bridge length. For our purposes, we combine this approximate model with MD and electronic structure computations of its parameters to screen candidate structures according to the estimated *k*_br_. The model is not a substitute for high-level non-adiabatic simulations that also include effects such as spontaneous TE localization and back-reactions from electronic to nuclear dynamics. These effects may reduce the value of *k*_br_ as compared to our estimate, but such simulations are very expensive for the purposes of initial screening.

**Figure 2:**
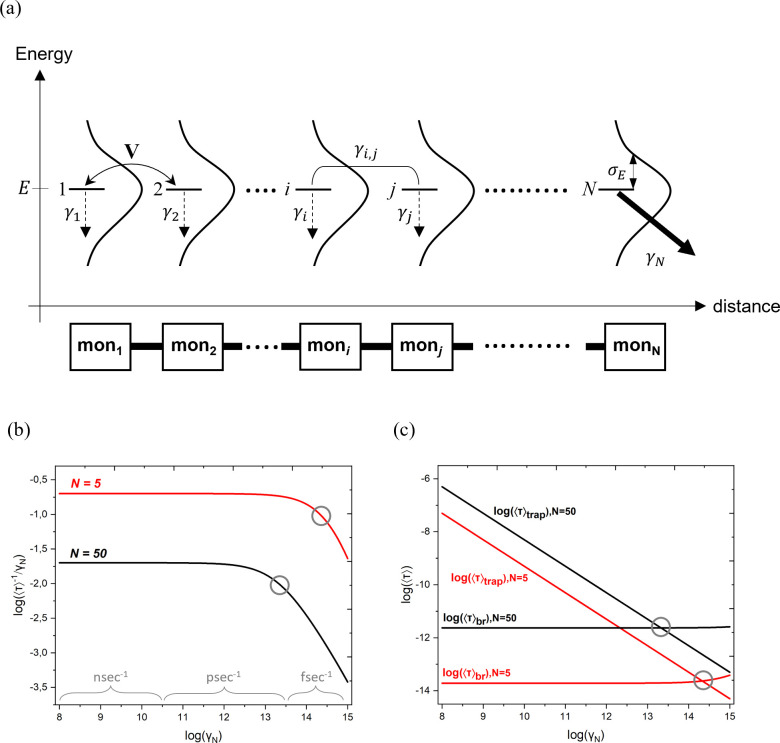
(a) Schematic diagram of the tight-binding model used to estimate the intrabridge TET rates *k*_br_ = ⟨τ⟩_br_^–1^ for a bridge with *N* sites (monomers). *E* is the monomer TE energy; *V* = *V*_rms_ is the rms nearest-neighbor TET coupling, and *σ*_*Ε*_ is the standard deviation of energies *E* arising from dynamic disorder. The *γ*_*i*_ values for *i* = 1 – *N* are the monomer TE population-relaxation rates. Each *γ*_*i*_ equals *γ*_*i*_ = *k*_B_^(ph)^ (monomer TE phosphorescence decay rate), and *γ*_*N*_ is the TET rate to an acceptor, *γ*_*N*_ ≈ *k*_B→A_^(TET)^. The γ*_i,j_* values are pure dephasing rates given by . (b) ⟨τ⟩^–1^/*γ*_*Ν*_ vs *γ*_*Ν*_ (both on a log_10_ scale) for *N* = 5 (red) and *N* = 50 (black) with *γ*_*i*_ = 1 μs^–1^ [*i* = 1 – (*N* – 1), ℏ*γ*_*i,j*_ = 0.1 eV, and *V* = 0.15 eV (*V* > *σ*_*Ε*_)]. (c) Trapping time ⟨τ⟩_trap_ and intrinsic bridge TET time ⟨τ⟩_br_ vs *γ*_*N*_, both on a logarithmic scale, for *N* = 5 and *N* = 50. The circles indicate the values of *γ*_*N*_ for which ⟨τ⟩_trap_ = ⟨τ⟩_br_.

The model allows for a precise definition of *k*_br_. If the TE is initially located at the first monomer [probability *P*_1_ (*t* = 0) = 1], the overall decay time of the TE is given by ⟨τ⟩ = ∑_*i*=1_^*N*^∫_0_^∞^ d*tP*_*i*_(*t*). Using the model described above, we compute ⟨τ⟩ numerically as a function of *N* and *γ*_*N*_ for the parameter regime *V* ≥ ℏ*γ*_deph_ ≈ 0.1 eV and for *γ*_*N*_ ranging from (1 fs)^−1^ to (10 ns)^−1^. We find that ⟨τ⟩ is not affected by the slow phosphorescence rates; i.e., we obtain identical results if we set *γ*_*i*__≠*N*_ = 0 and *γ*_*N*_ = *k*_B→A_^(TET)^. In addition, ⟨τ⟩ is given by the MFPT to *N*, i.e., ⟨τ⟩ = ∫_0_^∞^ d*ttP*_*N*_(*t*)/∫_0_^∞^ d*t P*_*N*_(*t*). The numerically calculated ⟨τ⟩ values are very close to the approximate formula^54^
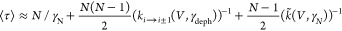
*k*_*i*__→*i*±1_(*V, γ*_deph_) = 2(*V*/ℏ)^2^/*γ*_deph_ is an effective transfer rate between nearest-neighbor TE populations and *k̃*(*V, γ*_*N*_) = 2(*V*/*ℏ*)^2^/*γ*_*N*_.^54^ We rewrite the analytical formula for ⟨τ⟩ as ⟨τ⟩ ≈ ⟨τ⟩_trap_ + ⟨τ⟩_br_, where we have separated the trapping time ⟨τ⟩_trap_ = *N*/*γ_Ν_* because it trivially depends on the bridge length and on the final site population-relaxation rate *γ*_*Ν*_. The remaining terms depend on the bridge Hamiltonian and the dephasing parameters and are written as ⟨τ⟩_br_ = ⟨τ⟩_br,deph_ + ⟨τ⟩_br,relax_, with  and . We see that when ⟨τ⟩_br_ ≪ ⟨τ⟩_trap_ the overall decay rate ⟨τ⟩^–1^ is independent of *V* and *γ*_deph_ because it is rate-limited by *γ*_*Ν*_ = *k*_B→A_^(TET)^, i.e., ⟨τ⟩^–1^ ≈ *γ*_*N*_/*N*. In the opposite limit, ⟨τ⟩_br_ ≫ ⟨τ⟩_trap_, the overall rate is given by ⟨τ⟩^–1^ ≈ ⟨τ⟩_br_^–1^. The intrabridge TET rate is given by *k*_br_ = ⟨τ⟩_br_^–1^.

Figure 2b shows the numerically computed ⟨τ⟩^–1^/*γ*_*N*_ versus *γ*_*N*_ on a logarithmic scale for *N* = 5 and *N* = 50 using *V* = 0.15 eV and ℏ*γ*_*i,j*_ = 0.1 eV. Figure 2c shows ⟨τ⟩_trap_ and ⟨τ⟩_br_ in logarithmic scale for the systems in Figure 2b. From the plots, we deduce that ⟨τ⟩_br_ = 10 fs and 0.1 ps for *N* = 5 and *N* = 50, respectively. The circles in both figures show the *γ*_*N*_ values for which ⟨τ⟩_br_ = ⟨τ⟩_trap_. For lower *γ*_*N*_ values, the overall rate ⟨τ⟩^–1^ becomes rate-limited by the trapping time. The computed values of ⟨τ⟩_br_ in Figure 2b suggest very fast TET for bridge lengths of approximately ∼1 nm (*N* = 5) and ∼15 nm (*N* = 50), respectively. The ultrafast transfer times for *N* = 5 (10 fs) should be compared to the much slower TET times of 100–200 ps for the dimer and trimer bridges in ref (47). The dependence of *k*_br_ as a function of bridge length *N* is given by (⟨τ⟩_br,deph_ + ⟨τ⟩_br,relax_)^–1^. In addition, for ℏ*γ*_deph_ ≤ *V*_rms_ and ℏ*γ*_deph_ ≈ 0.1 eV, it holds that ⟨τ⟩_br,deph_ ≫ ⟨τ⟩_br,relax_ for *γ*_*N*_ ≤ (10 fs)^−1^. In this broad regime, the distance dependence of the intrabridge TET rate is approximately  (see section 8 of the Supporting Information).

Due to the ultrafast *k*_br_ predicted for such types of bridges, a common situation for different choices of D and A will be that the TET D-to-B injection rate *k*_D→B_^(TET)^ and the B-to-A trapping rate *k*_B→A_^(TET)^ are slower than *k*_br_. Therefore, the effective (bridge-mediated) D-to-A TET rate *k*_D→A_^(eff,TET)^ will be rate-limited by the slowest of *k*_D→B_^(TET)^ and *k*_B→A_^(TET)^. If the initial D TE states are created by intersystem crossing (ISC) from D SE states produced by D photoexcitation, it is possible that fast D-to-A SET will take place. This is because any bridge architecture with a wide TE band will necessarily have at least an equally wide SE band. Thus, the SET transport channel may outcompete the TET channel. In this case, to enable D-to-A TET as opposed to SET, it is necessary to use donors with fast ISC rates as compared to the D-to-B SE injection rates, *k*_D_^(ISC)^ > *k*_D→B_^(SET)^.

As a case study of the constraints described above, we used a Ru(bpy)_3_^2+^ complex for D, due to its fast ISC rate (20–40 fs)^58,59^ and long triplet lifetime (∼10 μs).^60−62^ We connected it to a hexamer (IF6) bridge and a tetracene acceptor^63,64^ (Scheme 1D). The D and A moieties are linked to the bridge via methylene groups. Figure 3 (left-hand side) shows the TE eigenenergy manifold of the D–B–A system for one of the geometries we considered, computed at the gas phase using TD-DFT (ωB97/def2-SVP^65,66^ and see section 4 of the Supporting Information for higher-basis set computations). The right-hand side shows representative TE eigenstates of the entire D–B–A systems. The D-localized TE energies are above the lowest B-localized TE energies, the latter being above the lowest A-localized TE. In addition, there are no charge transfer (CT) D–B or A–B TE’s with energies below those of the other TE’s so that there is no CT state trapping. This is an optimal placement of the TE bands for coherent resonant D-to-A TET. In addition, the bridge-localized TE eigenstates have delocalization lengths that cover the entire bridge. For this system, the simulations described in Figure 2b for *N* = 6 predict an ultrafast intrabridge TET rate over a bridge length of ∼1.5 nm [*k*_br_ ≈ (10 fs)^−1^].

**Figure 3:**
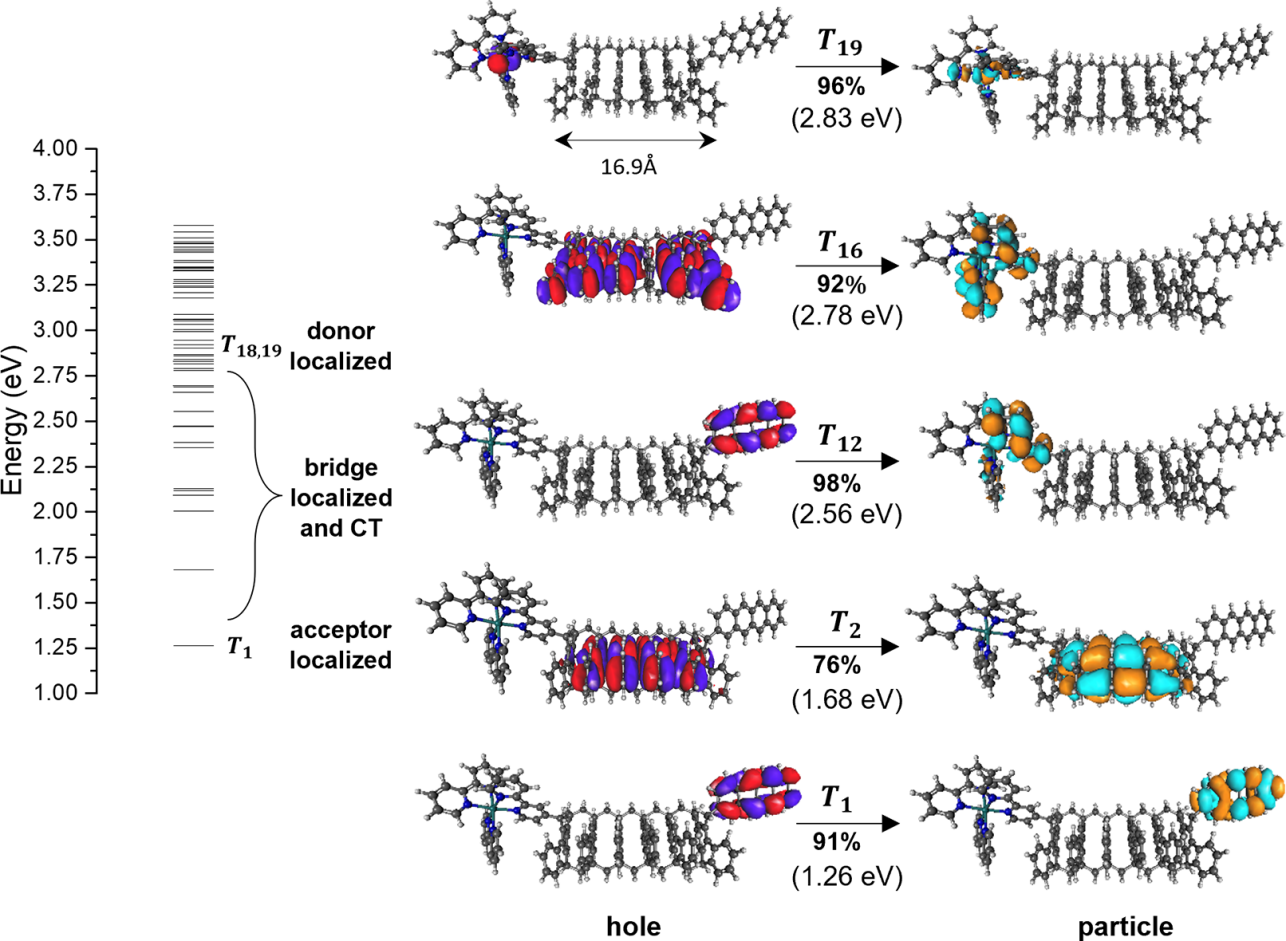
Energy level diagram (left) of the TE eigenstates of the Ru(bpy)_3_^2+^-IF6-Tet system computed at the ωB97/def2-SVP level of theory. Hole–particle pairs (right) of the natural transition orbitals (NTOs) of some TE’s. T_19_ is localized on D, T_1_ on A, and T_2_ on B, and T_16_ and T_12_ are D–B and D–A CT TE’s.

The design requirements for proposing the polymeric structure in Scheme 1 could be satisfied for a variety of monomer units. For example, for a perylene diimide (PDI) monomer, the inner-sphere reorganization energy of the lowest TE is small, approximately 0.1 eV (see section 6 of the Supporting Information). We performed computations on perylene-based polymers to explore different candidate structures (see section 6 of the Supporting Information). Among our trial systems, the best, from the point of view of optimizing coherent TET, are built from polymers with doubly linked monomers using methyl linkers that bring the monomers to sub-VdW intermonomer distances (see Figure 4). The necessity of double linkage is illustrated in Figure 4a–c. Figure 4a shows a dimer system with a single linker at C=O positions. This C=O to C–C substitution is challenging from a synthetic point of view, but it may be possible.^67^ If a single methyl bridge is used per monomer pair to build a polymer, MD simulations show that the π-stacking breaks for a long-enough polymer, diminishing the TET coupling and the TET efficiency (Figure 4b). Such a system can be transformed into a molecular wire that supports coherent TET over long distances if nearest-neighbor monomers are linked by two methyl groups as in Figure 4c. In this case, we find that *V*_rms_ ≈ 0.15 eV such that *V*_rms_ > *λ*^mon^/2, as in the previous IF example (see section 6 of the Supporting Information). Another good system for coherent TET that might be easier to synthesize compared to the previous one is shown in Figure 4d.^68^ For this dimer structure, we find that *V*_rms_ = 0.2 eV such that *V*_rms_ > *λ*^mon^/2.

**Figure 4:**
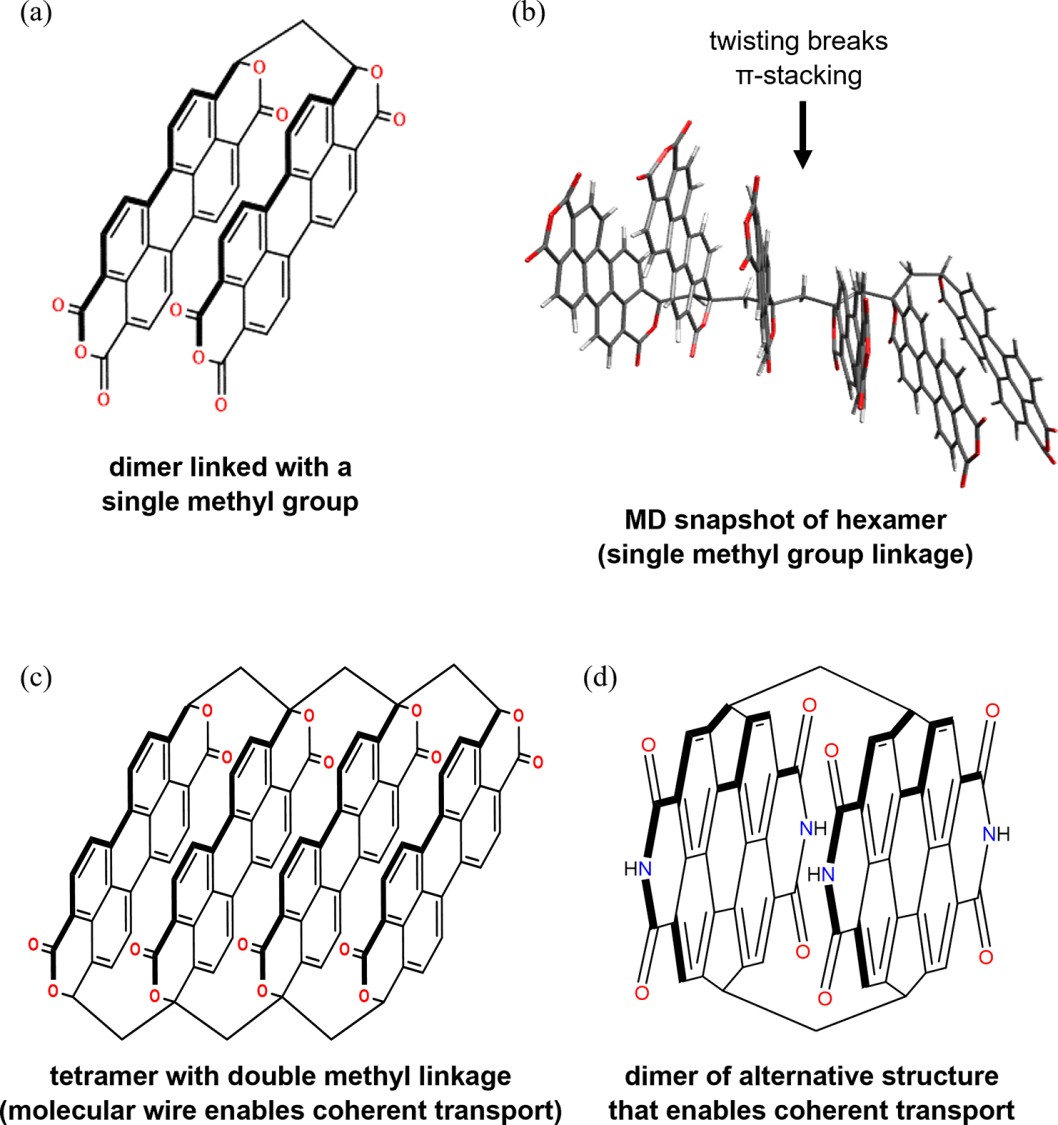
(a) Structure of the anthra[2,1,9-*def*:6,5,10-*d*′,*e*′,*f*′]diisochromene-1,3,8(10*H*)-trione dimer with a single methyl group linkage that enforces sub-VdW π-stacking. (b) Longer polymers with single methyl group linkages between monomers twist at room temperature, breaking the π-stacking and diminishing the interchromophore TET coupling. (c) This problem can be remedied by double methyl linkages between units of anthra[2,1,9-*def*:6,5,10-*d*′,*e*′,*f*′]diisochromene-1,3,8(3*H*,10*H*)-dione monomers. In this case, π-stacking is preserved for all pairs of nearest-neighbor monomers, and for each pair, *V*_rms_ ≥ σ_*E*_^mon^, *λ*^mon^/2. Such a wire supports long-distance coherent TET as in the case of the wires shown in Scheme 1. (d) Structure of two dicyclopenta[*ghi,pqr*]perylene derivatives linked with two methylene linkers to build a dimer. This type of structure also has strong TET coupling compared to the monomer reorganization energy (*V*_rms_ = 0.2 eV).

In conclusion, we have proposed design principles for building long and rigid molecular bridges with delocalized TE states at room temperature. Such bridges, when placed in nonpolarizable solvents, can mediate ultrafast and coherent TE transport from donor to acceptor moieties for distances that are much longer than what is currently possible. We have shown some example theoretical bridge structures that satisfy the design principles and are predicted to support single-molecule ultrafast and coherent TET. These structures are not meant to represent the only solutions to the constraints imposed by the design principles. They are shown because they minimize, at room temperature, intermonomer torsion and slide while simultaneously preserving sub-VdW intermonomer distances (the fluctuations in *V*^TET^ are at most 30% of the average). Although these features present a great challenge for synthetic chemistry, they are absolutely necessary for long-distance and ultrafast coherent TET along the molecular wire.

## Supporting Information Available

Summary of the computational methodologies for the IF*n* bridges; energy level diagrams of the SE’s and TE’s of the IF*n* bridges with *n* = 1, 2, 6, and 10; natural transition orbitals of the lowest TE states of the IF*n* bridges with *n* = 1, 2, 6, and 10; comparison calculations between CIS (TD-DFT) and higher-level *ab initio* methods for the TE energies of the IF dimer; testing the accuracy of the TE energies of the donor–bridge–acceptor systems using higher-level basis sets; computations of the IPR parameters; computations of SE and TE energy splittings of the perylene-based dimers; TET and SET couplings as a function of the interchromophore distance between two fluorene and two PDI monomers; computations of the distance dependence of the intrabridge TET rate; and ground state-optimized structures of the proposed IF*n* bridges with *n* = 2, 6, and 10 (PDF)
Transparent Peer Review report available (PDF)
