# Supplementary material for: Molecular Wires for Efficient Long-Distance Triplet Energy Transfer
Source: J Phys Chem Lett. 2022 Oct 10;13(41):9679–87. doi: 10.1021/acs.jpclett.2c02616 (PMC9589895; doi:10.1021/acs.jpclett.2c02616)
Supplement: Supplementary file 1 — jz2c02616_si_001.pdf [file jz2c02616_si_001.pdf]

Supplementary Material

Molecular Wires for Efficient Long-Distance Triplet Energy Transfer

Spyroulla A. Mavrommati and Spiros S. Skourtis*

Department of Physics, University of Cyprus, P.O. Box 20537, Nicosia 1678, Cyprus

*Electronic mail: skourtis@ucy.ac.cy

Contents

1 Summary of the computational methodologies for the IFn bridges **S2**

2 SE and TE states of the IFn bridges with n = 1,2,6,10 **S6**

3 Comparison between CIS (TD-DFT) and higher-level methods for the TE energies of the IF dimer **S12**

4 Testing the accuracy of the TE energies of the donor-bridge-acceptor systems using higher-level basis sets **S13**

5 Computation of the IPR parameters **S14**

6 SE and TE computations on the perylene-based dyads **S15**

7 TET and SET couplings as a function of the inter-chromophore distance **S18**

8 Distance dependence of the intra-bridge TET rate **S19**

9 Ground state structures of the proposed IF bridges **S20**

References **S28**

1 Summary of the computational methodologies for the IFn bridges

Ab-initio electronic structure calculations on the proposed bridges. We performed geometry optimizations on the singlet ground state of the indenofluorene (IF) monomer and the polymers (π-stacked dimer to hexamer). We used density functional theory (DFT) and the ORCA program package[1,2] at the B3LYP[3–5] level of theory in combination with the def2-TZVP[6] basis set. We applied the resolution of identity approximation (RI) for the Coulomb integrals and the chain of spheres approximation (COSX) to the exact exchange[7,8], in combination with the def2/J auxiliary basis sets[9] without symmetry constraints. Dispersion corrections were included via Grimme's D3 correction[10,11] using Becke-Johnson (BJ) damping[12]. The B3LYP functional paired with dispersion corrections was selected because it accurately predicts the equilibrium geometries and the interaction energies of van der Waals (VdW) complexes[13–15]. The ground-state geometry of the decamer was relaxed using steepest-descent molecular mechanics with the UFF (Universal Force Field) force field as implemented in the Avogadro software[16]. For these optimized structures we computed the lowest excited states (singlet-to-singlet and singlet-to-triplet transitions) using the Amsterdam Density Functional (ADF) program package[17], and time-dependent DFT (TD-DFT) with the Tamm-Dancoff approximation (TDA)[18]. We used the M06-2X functional[19,20], combined with DZP basis set[21] without symmetry constraints. Meta-hybrids reliably predict the excitation energies and spectroscopic properties of non-covalent complexes[22]. The singlet-to-triplet transitions were also computed with the Configuration Interaction Singles (CIS) method, as implemented in the ORCA program package, in combination with the def2-SVP basis set[23,24].

In section 2 we show the energies of the singlet exciton (SE) and triplet exciton (TE) eigenstates of the monomer, dimer, hexamer and decamer bridges. Figure S3 shows the SE energies computed at the M06-2X/DZP level of theory while figures S4 and S5 show the TE energies computed at the M06-2X/DZP and CIS/def2-SVP levels, for comparison. In addition, tables S1, S2, S3 and S4 show the delocalization of the molecular orbitals that contribute to the lowest two TE eigenstates, of the monomer, dimer, hexamer and decamer, computed with CIS/def2-SVP (M06-2X/DZP method gives similar delocalization of the orbitals).

Reorganization energy calculations for the monomer. For the computations of the reorganization energies of the monomer, we used ADF to diagonalize the mass-weighted Hessian matrix $\tilde{H}$ by $L^T\tilde{H}L = \omega^2$ on the optimized structures of the monomer for the ground state (S_0) and first TE state (T_1), in order to obtain the normal modes and their frequencies (where L denotes the eigenvectors and ω the eigenvalues)[25,26]. Then we used the FCF (Franck-Condon factors) auxiliary program as implemented in ADF to compute the reorganization energy for each normal mode[27,28]. The total reorganization energy for the transition $S_0 \rightarrow T_1 (T_1 \rightarrow S_0)$ equals to the sum of the normal mode reorganization energies. The geometry optimization and frequency computations employed the BP86 functional[4] and the TZ2P basis set[21] with the TDA. Figure S1 shows schematically the monomer reorganization energies (λ_D and λ_A) used to compute

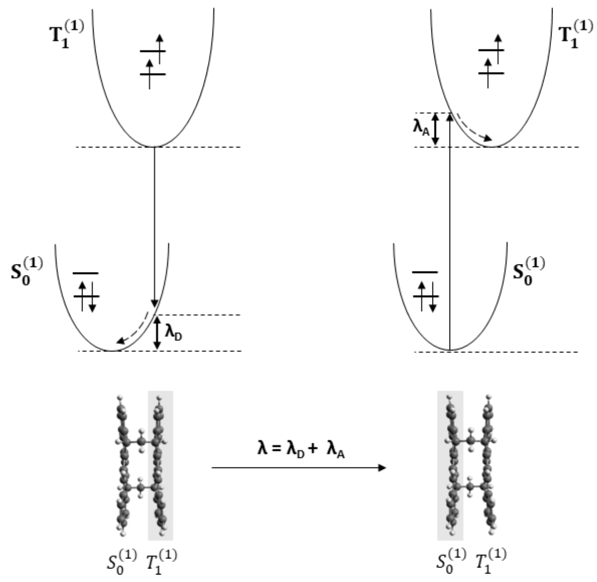

Figure S1. Schematic representation of the potential energy surfaces (PES) of the singlet ground state $S_0^{(1)}$ and the first TE state $T_1^{(1)}$ of the donor and acceptor moieties (i.e., IF monomer). The donor-to-acceptor reorganization energy λ, is the sum of the reorganization energies of the donor and acceptor,according to eq. S1.

the activation energy U^{act} in the main text. In the classical high-temperature limit, the total reorganization energy (λ) for donor (D) to acceptor (A) TET is approximated by (see pages 289-291 in ref. 29),

$$\lambda = \lambda_D + \lambda_A. \qquad \text{(eq. S1)}$$

Ab-initio electronic structure calculations on the donor-bridge-acceptor complexes. We performed excited-state computations (with TD-DFT) on the combined donor-bridge-acceptor system using the ORCA program package and with the ωB97 range-separated functional[30], in combination with the TDA and without symmetry constraints. Range-separated functionals reproduce well the optical absorption spectrum and the lowest-lying triplet excited states of heavy-metal systems (such as $Ru(bpy)_3^{2+}$)[31–33]. In addition, range-separated functionals combined with TDA perform well in the calculation of TE energies in long π-conjugated molecules and accurately predict the charge-transfer (CT) donor-bridge and bridge-acceptor excitations[34–37]. The def2-SVP basis set was used for all the atoms. In the case of Ru(II), the def2-SVP basis set was used for the outer-core $[(4s)^2(4p)^6]$ and the valence $(4d)^6$ electrons while the inner-core electrons were treated as effective core potential (ECP)[38]. We also performed computations with the CIS method and the def2-SVP basis set for comparison. The results were found to be similar to those computed with TDA, i.e., the ordering of the TE exciton eigenergies and the localization of the TE eigenstates were similar for both methods.

Effects of thermal fluctuations on the polymer structural stability. To test the structural sta-

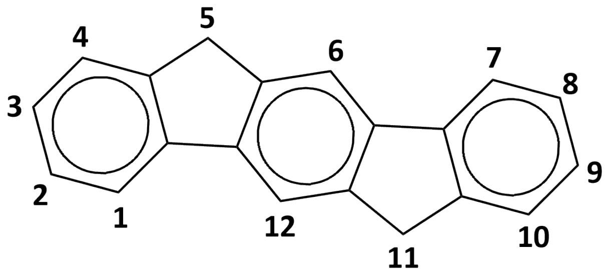

Figure S2. The IF monomers are cut off from the dimer and each monomer is capped with hydrogens at positions 5' and 11'.

bility of the polymers (dimer up to decamer), we performed classical molecular dynamic (MD) simulations using the AMBER program package[39]. The optimized structures of the IF bridges were used as the initial structures for the simulations. The atomic charges were calculated with the AM1-BCC model[40,41] and the simulations were carried out under conditions of constant temperature (298 K) and the GAFF force field[42], for 10 nsec using 1 fsec integration step and the Langevin thermostat with frequency of collision[43–45] equal to 5 $(\mathrm{ps})^{-1}$.

Effects of thermal fluctuations on the nearest-neighbor TE transfer (TET) coupling. The effects of conformational fluctuations on the TET coupling were tested by performing *ab-initio* MD simulations on the dimer structure, using the Q-CHEM program package[46]. For the computations we used the B3LYP functional with the 6-31G* basis set[47] in combination with Grimme's D3BJ dispersion correction. The simulation was carried out with the NVT (constant volume and temperature) ensemble using the white noise Langevin thermostat[48] at room temperature (298 K) for a total of 23 psec with 1 fsec time step. The system was equilibrated during the first 3 psec. The 20 psec MD simulation time for the dimer is sufficient for sampling its vibrational motions (we performed normal mode computations on the dimer using ADF and BP86/TZ2P and found that the lowest-frequency normal mode has a period of approximately 1 psec). To compute the fluctuations of the TET coupling we picked 190 MD snapshots separated by 100 fsec and for each snapshot we computed the TE splitting between the lowest two TE states of the dimer $\left(\Delta E = E_{T_2^{(2)}} - E_{T_1^{(2)}} = 2V^{TET}\right)$ using the ORCA program at CIS/def2-SVP level of theory. From the collection of the MD-derived splittings we deduced the root mean square (rms) of the TET coupling (V_{rms} in the main text).

Effects of thermal fluctuations on the monomer TE energies. To compute the fluctations of the monomer TE energies we used the abovementioned MD-derived dimer structures to create 380 (190 × 2) monomer structures by cutting the two methylene bridges, and capping the carbons at the positions 5´and 11´ with hydrogen atoms (see figure S2). We computed the energy of the lowest TE state of each monomer structure at the CIS level (in combination with def2-SVP basis set) using the ORCA program. From the collection of TE energies we estimated the standard deviation σ_E^{mon} of the main text.

Effects of structural fluctuations on the TE delocalization. We studied the effects of thermal fluctuations of the molecular orbitals of the IF decamer (IF10) by performing classical MD simulations and using the MD-derived snapshots for subsequent computations of the frontier molecular orbitals. We used the AMBER and the ORCA program packages to perform the MD simulations and the quantum chemical calculations respectively. First, the system was equilibrated for 1 nsec using the Langevin thermostat (frequency of collision equals to 5 $(\mathrm{ps})^{-1}$) at a temperature of 298 K with a time step of 1 fsec. After equilibration, we ran MD simulations up to 9 nsec using 1 fsec time step, with the GAFF force field. The atomic charges were calculated using the AM1-BCC charge model. We performed computations of the molecular orbitals on 3000 MD trajectories separated by 3 psec. These calculations were carried out at HF/def2-SVP level of theory and the orbital eigenstates were used to evaluate the inverse participation ratio (IPR) of the orbitals involved in the lowest TE's of the decamer (see section 5 for the IPR).

2 SE and TE states of the IFn bridges with n = 1,2,6,10

In this section we show the computed SE and TE eigenenergies of the proposed bridges (monomer, dimer, hexamer, decamer). In figures S4 and S5 we compare the performance of the M06-2X functional to the CIS method for the computation of TE energies. The results show that the TE energies (especially the lowest-lying TE's) and splittings are similar. Tables S1, S2, S3 and S4 show the natural transition orbitals (NTOs)[49] of the lowest TE's of the monomer, dimer, hexamer and decamer, computed at the CIS/def2-SVP level of theory. The last column of each table shows the transition amplitudes of particle-hole excitations (in percentages).

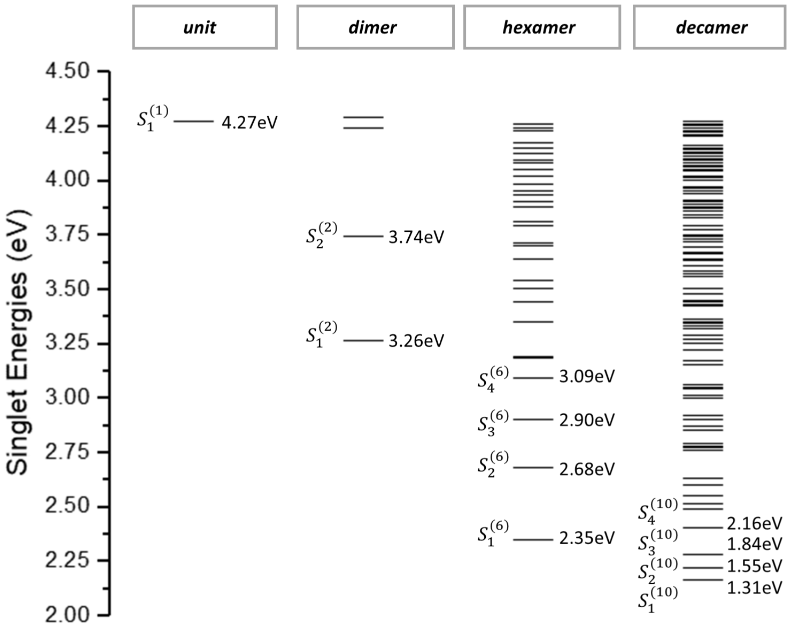

Figure S3. SE states ($S^{(n)}$) of the IFn bridges (n = 1,2,6,10) computed at the M06-2X/DZP level of theory using the ADF program package. The notations (1), (2), (6), (10) denote monomer, dimer, hexamer and decamer systems respectively.

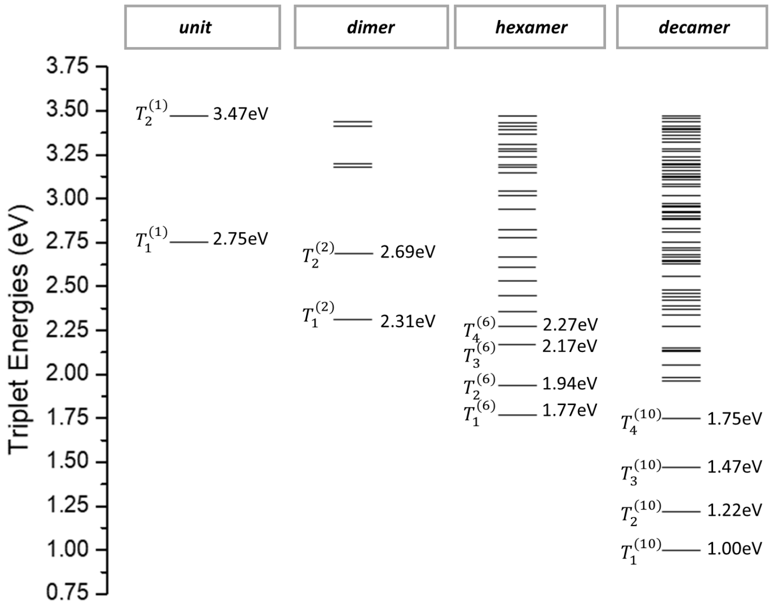

Figure S4. TE states ($T^{(n)}$) of the IFn bridges (n = 1,2,6,10) computed at the M06-2X/DZP level of theory using the ADF program package. The notations (1), (2), (6), (10) denote monomer, dimer, hexamer and decamer systems respectively.

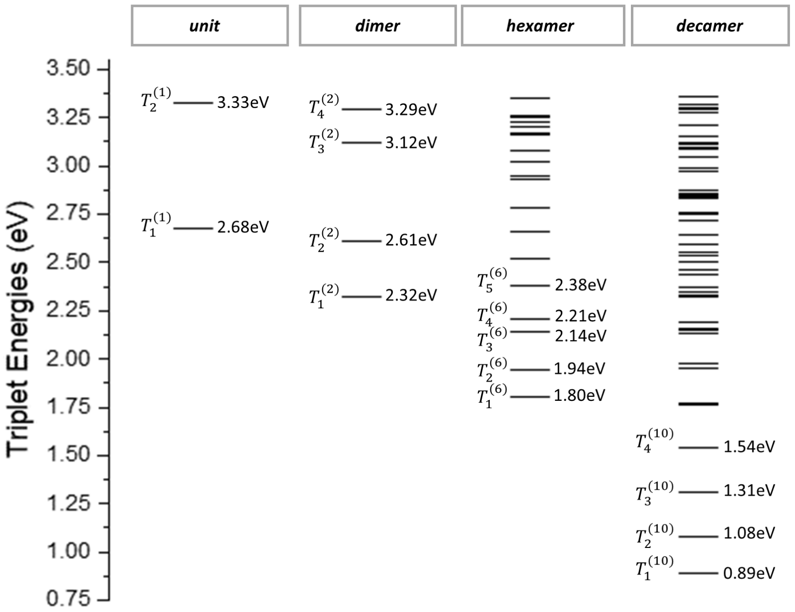

Figure S5. TE states ($T^{(n)}$) of the IFn bridges (n = 1,2,6,10) computed at the CIS/def2-SVP level of theory using the ORCA program package. The notations (1), (2), (6), (10) denote monomer, dimer, hexamer and decamer systems respectively.

Table S1. Hole-particle pairs of NTOs of the lowest two ($T_1^{(1)}$, $T_2^{(1)}$) TE states of the monomer, computed at the CIS/def2-SVP level of theory (based on a 0.02 isosurface value).

Triplet State	hole	particle	contribution
1	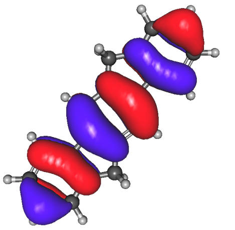	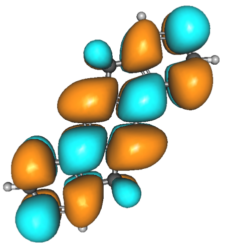	62%
2	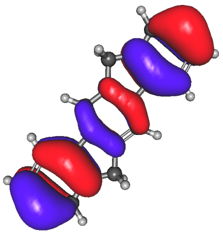	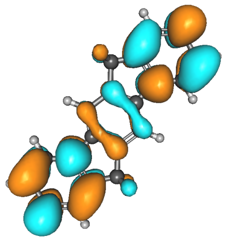	27%
	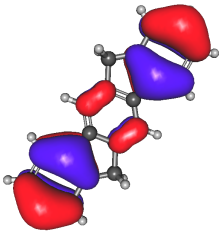	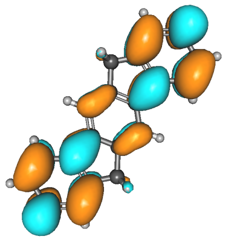	27%

Table S2. Hole-particle pairs of NTOs of the lowest two ($T_1^{(2)}$, $T_2^{(2)}$) TE states of the dimer, computed at the CIS/def2-SVP level of theory (based on a 0.02 isosurface value).

Triplet State	hole	particle	contribution
1	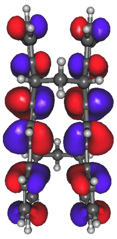	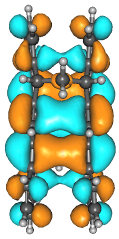	52%
	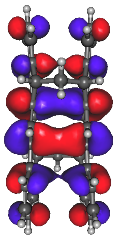	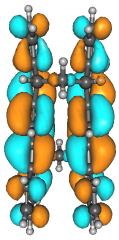	15%
2	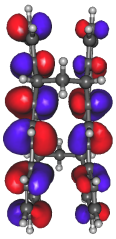	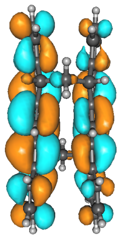	32%
	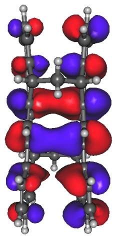	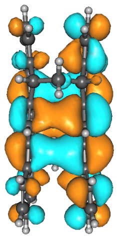	31%

Table S3. Hole-particle pairs of NTOs of the lowest two ($T_1^{(6)}$, $T_2^{(6)}$) TE states of the hexamer, computed at the CIS/def2-SVP level of theory (based on a 0.01 isosurface value).

Triplet State	hole	particle	contribution
1	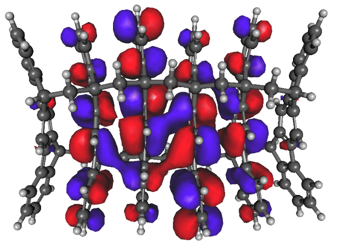	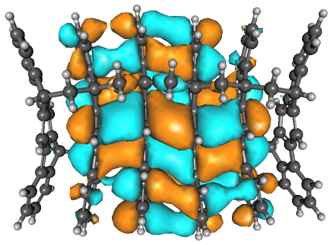	43%
	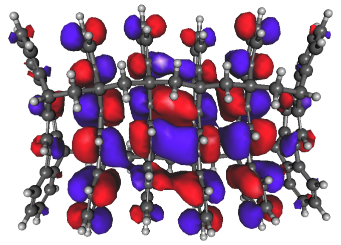	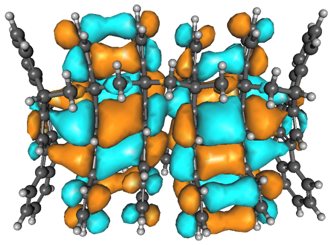	15%
2	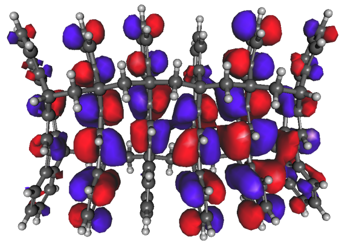	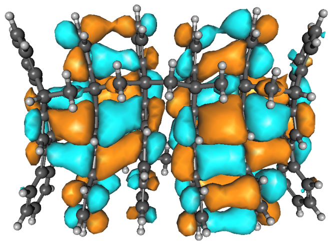	30%
	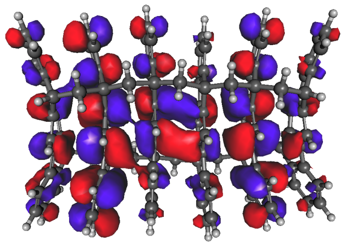	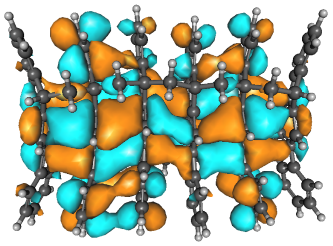	26%

Table S4. Hole-particle pairs of NTOs of the lowest two ($T_1^{(10)}$, $T_2^{(10)}$) triplet states of the decamer, computed at the CIS/def2-SVP level of theory (based on a 0.005 isosurface value).

Triplet State	hole	particle	contribution
1	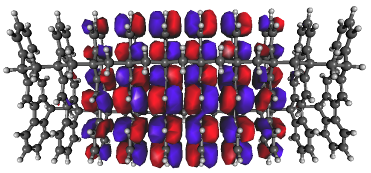	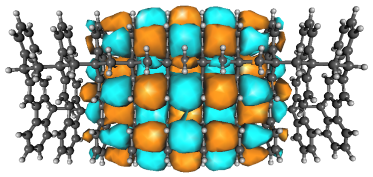	52%
	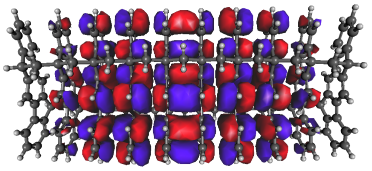	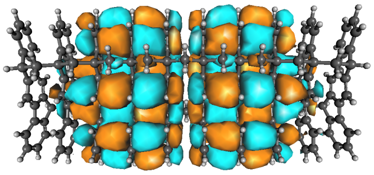	15%
2	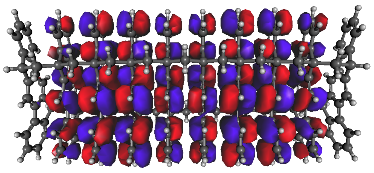	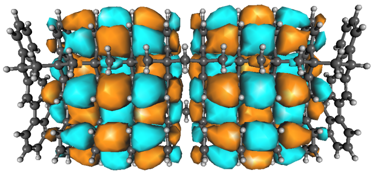	33%
	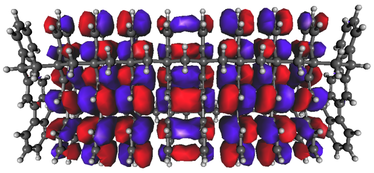	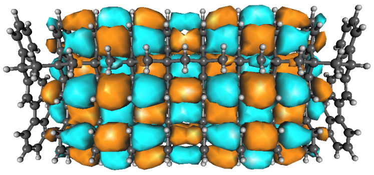	29%

3 Comparison between CIS (TD-DFT) and higher-level methods for the TE energies of the IF dimer

The lowest TE states of the IF dimer were computed using high-level *ab-initio* methods including similarity-transformed equation of motion coupled cluster method (STEOM-CC)[50–55], perturbative doubles correction for the single excitation configuration interaction method (CIS(D))[56], and algebraic diagrammatic construction method to second-order (ADC(2))[57,58]. We computed the lowest two TE states $\left(T_1^{(2)}, T_2^{(2)}\right)$ of the dimer using these methods and we compared the results with those given by TD-DFT and CIS methods.

The STEOM-DLPNO-CCSD (similarity transformed equation of motion − domain-based local pair natural orbital − coupled cluster with singles and doubles excitations) were carried out using the ORCA program package in combination with def2-SVP basis set. The $T_{CutPNOsingles}$ keyword was set to $1e-11$ and the active selection keywords "Othresh"and "Vthresh"were set to 0.005. The CIS(D) and ADC(2) methods were both carried out using the Q-CHEM program package. The CIS(D) method was combined with def2-SVP basis set and the ADC(2) with 6-31G. All the computations were performed at the optimized geometry of the dimer (see section 1).

Table S5. TE energies of the IF dimer computed with different *ab-initio* methods. Values are in eV.

	**STEOM-DLPNO-CCSD** (eV)	**CIS(D)** (eV)	**ADC(2)** (eV)	**CIS** (eV)	**M06-2X** (eV)	**ωB97** (eV)
$T_1^{(2)}$	2.20	3.12	2.89	2.32	2.34	2.82
$T_2^{(2)}$	2.55	3.52	3.32	2.61	2.70	3.18
ΔE	0.35	0.40	0.43	0.29	0.36	0.36

Our results show that, although the absolute TE energies vary from 2.2 eV to 3.1 eV across methods, the energy splitting ΔE ($\Delta E = E_{T_2^{(2)}} - E_{T_1^{(2)}}$) that is used to estimate the TET coupling varies much less 0.3 eV to 0.4 eV.

4 Testing the accuracy of the TE energies of the donor-bridge-acceptor systems using higher-level basis sets

In this section we test the accuracy of the TD-DFT computations on the donor-bridge-acceptor system, with respect to the chosen basis set. To reduce computational cost we performed our computations on the donor-bridge system where the bridge is the IF2. We used the ωB97 functional in combination with the def2-SVP basis set of double-zeta quality. These results were compared to those performed with the def2-TZVP basis set which is of triple-zeta quality. The results are summarized in table S6.

Table S6. Comparison of TE energies computed using the ωB97 functional with the def2-SVP and def2-TZVP basis set for the Ru(bpy_3^{2+})-IF2 system. Energy values are in eV.

Triplet state	def2-SVP	def2-TZVP	% deviation
1	2.46	2.43	1.15
2	2.51	2.48	1.29
3	2.52	2.49	1.24
4	2.60	2.57	1.25
5	2.64	2.60	1.58
6	2.66	2.61	1.80
7	2.69	2.66	1.13
8	2.99	2.95	1.29
9	3.07	3.04	0.82
10	3.10	3.07	0.85
11	3.16	3.12	1.19
12	3.21	3.19	0.56
13	3.23	3.20	0.97
14	3.31	3.30	0.52
15	3.40	3.35	1.46
16	3.41	3.36	1.31
17	3.43	3.39	1.36
18	3.46	3.40	1.59
19	3.48	3.45	0.78
20	3.51	3.48	0.72

The results on the lowest 20 triplet excited states show that the TE energies computed with def2-SVP are systematically larger by approximately 2% as compared to those computed with def2-TZVP. Also the ordering of the TE states does not change. Therefore, for our calculations on the donor-bridge-acceptor systems we used the def2-SVP basis set since it provides accurate results with lower computational time.

5 Computation of the IPR parameters

The delocalization of molecular wavefunctions is described by the inverse participation ratio (IPR)[59–64]. Consider the kth molecular orbital wavefunction,

$$|\chi_k\rangle = \sum_{n=1}^{N} c_n^k \, |\varphi_n\rangle \qquad \sum_{n=1}^{N} \mid c_n^k \mid^2 = 1 \tag{eq. S2}$$

expressed in the basis of N atomic (site) orbitals $(\varphi_1, \varphi_2, ...\varphi_N)$. The IPR for this molecular orbital is defined as,

$$L_k = \left(\sum_{n=1}^{N} \mid c_n^k \mid^4 \right)^{-1} \tag{eq. S3}$$

where c_n^k is the amplitude of the kth eigenfunction corresponding to the nth site. The IPR can range from a value of 1 for a fully localized molecular orbital (localized at a single φ_n), to a value of N for a fully delocalized molecular orbital.

The TE states $(|\Upsilon_T\rangle)$ are linear combinations of singly excited slater-type determinants that describe the promotion of an electron from an occupied molecular orbital χ_a to a virtual molecular orbital χ_i,

$$|\Upsilon_T\rangle = \sum_{a,i} b_{a,i} |\Psi_a^i\rangle. \tag{eq. S4}$$

The TE delocalization can be estimated from the delocalization length of the occupied and virtual molecular orbitals involved in the excitation (L_a, L_i).

We computed the IPR's for the HOMO, HOMO-1 and LUMO, LUMO+1 molecular orbitals of the decamer since they contribute to the lowest TE state ($T_1^{(10)}$: 40% HOMO⟶LUMO and 10% HOMO-1⟶LUMO+1 in figure 1(b) of the main text). The reference values for the IPRs are those computed for the optimized geometric conformation of the decamer (shown in figure 1(b) of the main text). We found that the IPRs for the HOMO and HOMO-1 orbitals (figure 1(b), main text) are equal to 336 and 401 respectively, and those for the LUMO and LUMO+1 orbitals (figure 1(b), main text) are equal to 489 and 462 respectively. These results reveal that, for the decamer bridge, an IPR value of the order of $\sim$ 400 (average value of the four molecular orbitals that contribute to the lowest TE state) describes a fully delocalized TE state. We also computed MD-averaged IPR values $\bar{L}_k$ and standard deviations σ_{L_k} for the relevant molecular orbitals χ_k contributing the TE state (using 3000 MD snapshots). The values $\bar{L}_k \pm \sigma_{L_k}$ for the HOMO, HOMO-1, LUMO and LUMO+1 orbitals are 207 ± 56, 258 ± 65, 335 ± 63 and 365 ± 54, respectively (mean value: 292 ± 60). Thus, the MD-averaged IPR is approximately 73% of the reference IPR value for the optimized geometry of figure 1(b). These results imply that thermal fluctuations do not affect the delocalization of the TE's on longer polymers.

6 SE and TE computations on the perylene-based dyads

Figure S6 shows the π-stacked perylene-based dimer structures we used to compute SET and TET couplings. These are: perylene-3,4:9,10-bis(dicarboxyimide) (PDI) ((a) and (b)), perylene-3,4-dicarboximide (PMI) (c) and terrylene-3,4:11,12-bis(dicarboximide) (TDI) (d).

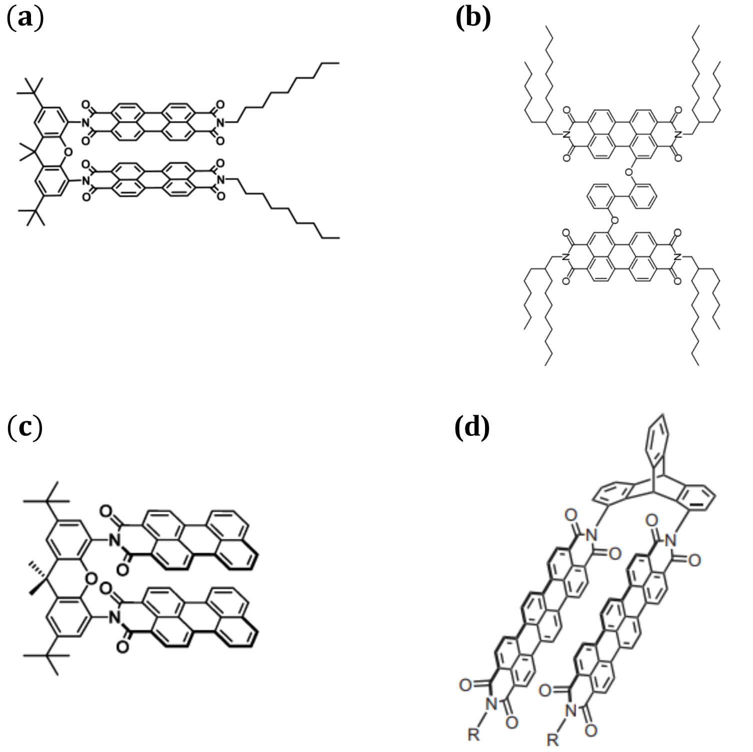

Figure S6. Molecular structures of perylene-based dimers. (a) PDI dimer of ref. 65, (b) PDI dimer of ref. 66, (c) PMI dimer of ref. 67 and (d) TDI dimer of ref. 68 with R = Me.

Table S7 summarizes the lowest two SE and TE energies of the dimers and the energy splittings: $\varDelta E_{SE} = E_{S_2^{(2)}} - E_{S_1^{(2)}} = 2V^{SET}$ and $\varDelta E_{TE} = E_{T_2^{(2)}} - E_{T_1^{(2)}} = 2V^{TET}$, where the notation (2) denotes dimer. The excited-state computations were performed using the ORCA program package at the CIS/def2-SVP level and also at the ωB97/def2-SVP level applying the TDA. The geometric structures (a), (c) and (d) (see figure S6), were taken from refs 65, 67 and 68 respectively. The ground-state structure of (b) was built using the Avogadro software, and optimized at the B3LYP/def2-SVP level of theory (including dispersion corrections D3BJ) using the ORCA program.

The experimentally-derived SET couplings (from absorption band widths)[69] are approximately 0.1 eV. Further, SET couplings for several organic π-stacked dimeric systems computed using high-level methods are 0.1 - 0.4 eV[37,66,70]. Our CIS and DFT computations on the π-stacked dimeric systems of figure S6 give SET coupling values consistent with the abovementioned SET coupling magnitudes (table S7). The TET couplings computed by both DFT and CIS methods

are similar to each other and are much weaker than the SET couplings (see table S7). On the other hand, for the proposed IF dimer the TET coupling is an order of magnitude greater than those predicted for the perylene-based dimers.

Table S7. SE and TE energy splittings (ΔE_{SE}, ΔE_{TE}) of the perylene-based dimers (shown in figure S6) and the IF dimer. Values are in eV.

	**(a)**		**(b)**		**(c)**		**(d)**		**IF**	
	**CIS**	**ωB97**	**CIS**	**ωB97**	**CIS**	**ωB97**	**CIS**	**ωB97**	**CIS**	**ωB97**
ΔE_{SE}	0.39	0.40	0.20	0.17	0.25	0.26	0.39	0.35	0.74	0.53
ΔE_{TE}	0.02	0.03	0.02	0.03	0.01	0.02	0.05	0.05	0.29	0.35

The inter-chromophore distance between the monomers in the above-mentioned dimers (figure S6) is on average greater than VdW ($\sim$ 4Å). To reduce this distance we tried to link several perylene-based monomers via shorter bridges as shown in figures S7 and figure 4 in the main text. Similar monomer structures to the ones shown in the figures have already been synthesized, e.g., see refs. 71,72. We performed MD computations to estimate the V_{rms} in these systems. The results show that the structure in figure S7(a) is very rigid (very low torsional and slippage motions), but the intermonomer distance is always greater than $\sim$ 4Å. This causes weak TET coupling of the order of $\sim$ 0.01 eV. The structure shown in figure S7(b) shows large dynamic disorder that diminishes the TET coupling ($\sim$ 0.03 eV). On the other hand, the structure shown in figure 4(c) in the main text exhibits large interchromophore TET coupling because it maintains tight $\pi-$stacking. The average value of the TET coupling equals to $V_{ave} = 0.14$ with $\sigma_V = 0.05$ eV and the rms coupling equals to $V_{rms} = 0.15$ eV. If the monomers are linked with a single methyl group (see figure 4(a) in the main text), MD simulations on the dimer and longer polymers (i.e., hexamer) show that the $\pi-$stacking breaks (figure 4(b)). Therefore, to prevent this breaking it is necessary to link the nearest-neighbor chromophores with methylene bridges in two positions (as shown in figure 4(c) in the main text). We also computed the TE reorganization energy of the perylene-based monomer whose dimer structure is shown in figure 4(a) (λ computed as described in section 1). We found that $\lambda_{D(A)} = 0.14$ eV such that $U^{act} = 0.07$ eV. Therefore for these types of doubly-linked bridges, $V_{rms} > \lambda/2$. Another candidate that also looks promising for building molecular wires that support coherent TET is shown in figure 4(d) in the main text. For this system, the MD simulations followed by CIS computations give $V_{rms} = 0.2$ eV, i.e., $V_{rms} > \lambda/2$.

The MD simulations on the dimers and the longer polymers were performed using the AMBER program package for a total of 10 nsec with 1 fsec time step and the NVT ensemble at a temperature of 298 K (Langevin thermostat with frequency of collision equal to 5 $(\text{ps})^{-1}$). To estimate the MD-averaged TET coupling, we performed quantum chemical calculations on 2000 MD-snapshot structures at the CIS/def2-SVP level of theory (using ORCA).

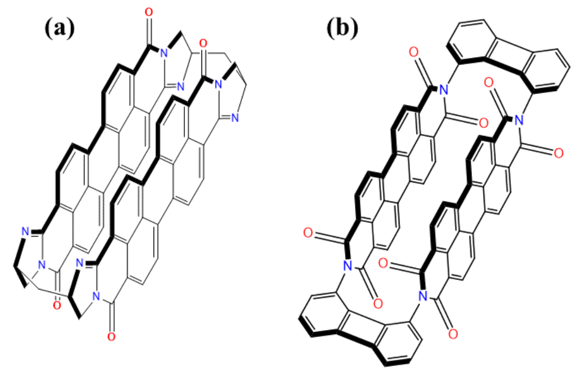

Figure S7. Molecular structures of perylene-based chromophore dimers that were tested for coherent TE transport. (a) Bisbenzimidazo[2,1-a:2',1'-a']anthra[2,1,9-def:6,5,10-d'e'f']diisoquinoline-10,21-dione monomers linked with methyl groups, (b) anthra[2,1,9-def:6,5,10-d'e'f']diisoquinoline-1,3,8,10(2H,9H)-tetraone (perylene diimide) monomers linked with biphenylene bridges.[71]

7 TET and SET couplings as a function of the inter-chromophore distance

We tested the TET and SET couplings as a function of the inter-chromophore distance between two fluorene monomers and two PDI monomers (3,4,9,10-Perylenetetracarboxylic dianhydride), as shown in figure S8. The couplings were computed using the ORCA program package at CIS/def2-SVP level. Our results reveal that the TET coupling becomes two to three orders of magnitude smaller than the SET couplings when the distance between the monomers becomes greater than VdW.

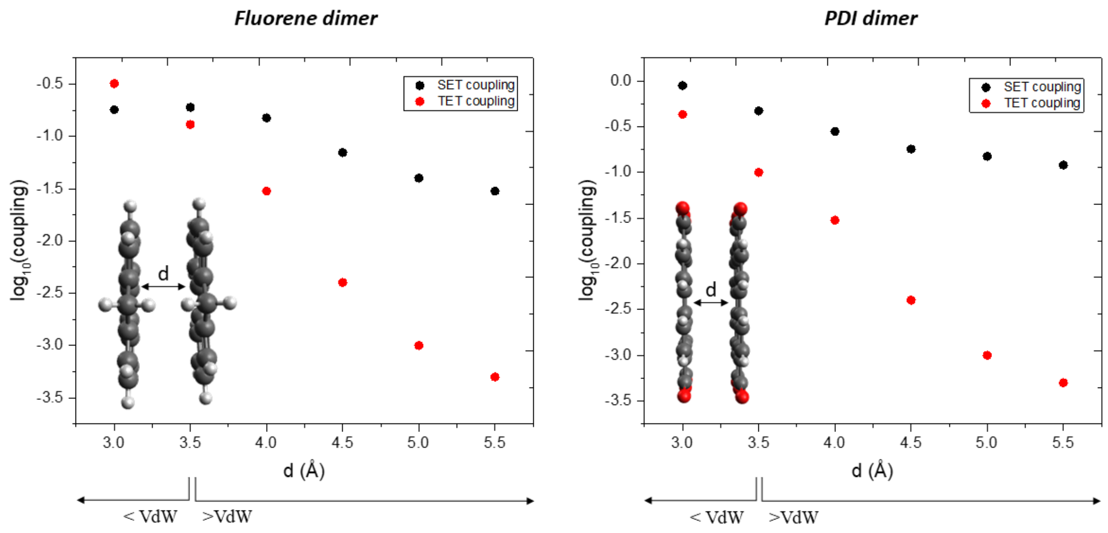

Figure S8. SET and TET couplings (in logarithmic scale) versus distance (in Angstroms). The TET coupling drops considerably below 0.10 eV for distances greater than VdW while the SET coupling is much greater. *left.* Computations on the fluorene dimer, *right.* Computations on the PDI dimer.

8 Distance dependence of the intra-bridge TET rate

From the analytical solutions to the Mean First Passage Time (MFPT) in the model described in ref. 73, we define the intra-bridge rate as $k_{br} = 1/\langle\tau\rangle_{br}$ where,

$$\langle\tau\rangle_{br} \approx \langle\tau\rangle_{br,deph} + \langle\tau\rangle_{br,relax}$$

$$\langle\tau\rangle_{br,deph} = \frac{N(N-1)}{2}\frac{\gamma_{deph}}{2(V/\hbar)^2} \quad \text{(eq. S5)}$$

$$\langle\tau\rangle_{br,relax} = \frac{N-1}{2}\frac{\gamma_N}{2(V/\hbar)^2}.$$

Figure S9 shows the intra-bridge TET rate k_{br} (in logarithmic scale) as a function of the bridge length N, computed for $V = 0.2$ eV, $\hbar\gamma_{deph} = 0.1$ eV and $\gamma_N = (10 \text{ nsec})^{-1}$. In the range of $\gamma_N \leq (10 \text{ fsec})^{-1}$ the behavior of k_{br} is near identical and is dominated by $\langle\tau\rangle_{br,deph}$.

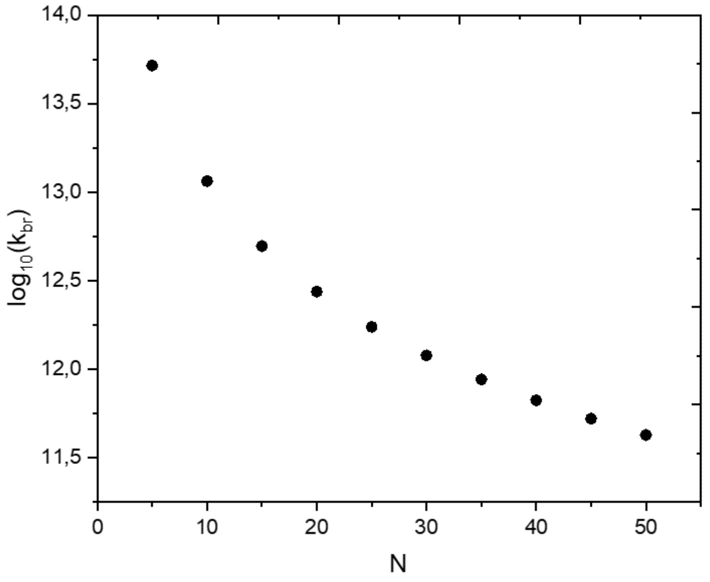

Figure S9. Distance dependence of the intra-bridge TET rate (k_{br}) (in logarithmic scale) as a function of the length of the bridge. The simulations were performed for $V = 0.2$ eV, $\hbar\gamma_{deph} = 0.1$ eV and $\gamma_N = (10 \text{ nsec})^{-1}$.

9 Ground state structures of the proposed IF bridges

Below are the atomic coordinates of the optimized ground-state structures computed according to section 1.

<u>IF dimer:</u>

C	−2.389	1.438	−0.054
C	−2.498	0.232	−0.743
C	−3.628	−0.566	−0.599
C	−3.406	1.860	0.801
C	−4.526	1.065	0.958
C	−4.643	−0.142	0.251
C	−5.760	1.294	1.815
C	−6.642	0.122	1.418
C	−5.932	−0.750	0.571
C	−6.453	−1.995	0.228
C	−7.706	−2.325	0.704
C	−7.946	−0.159	1.787
C	−8.487	−1.384	1.402
H	−1.505	2.050	−0.180
H	−1.695	−0.087	−1.395
H	−3.713	−1.499	−1.143
H	−3.304	2.789	1.348
H	−6.237	2.232	1.507
H	−5.866	−2.700	−0.348
H	−8.516	0.530	2.400
C	−9.816	−3.279	1.131
C	−9.814	−1.964	1.624
C	−10.971	−1.415	2.163
C	−12.134	−2.179	2.180
H	−13.042	−1.763	2.597
C	−12.142	−3.470	1.661
H	−13.057	−4.049	1.672
C	−10.977	−4.029	1.139
H	−10.986	−5.042	0.754
H	−10.978	−0.407	2.556
C	−8.432	−3.654	0.641
H	−8.481	−4.020	−0.389
H	−11.539	−3.273	6.199
H	−11.724	−5.411	4.984
C	−10.737	−3.588	5.544
C	−10.844	−4.795	4.856
C	−9.611	−2.786	5.397
C	−9.827	−5.213	3.998
H	−9.528	−1.853	5.941
H	−9.927	−6.143	3.451
C	−8.596	−3.206	4.545
C	−8.710	−4.414	3.838
H	−6.998	−5.575	3.283
C	−7.310	−2.595	4.222
C	−7.478	−4.638	2.978
H	−7.379	−0.645	5.143
C	−6.791	−1.349	4.565
C	−6.598	−3.465	3.375
H	−4.768	0.680	5.186
C	−5.538	−1.016	4.090
C	−5.295	−3.182	3.006
C	−4.814	0.313	4.155
C	−4.755	−1.956	3.393
H	−4.723	−3.870	2.393
C	−3.427	−0.061	3.670
C	−3.428	−1.376	3.175
C	−2.266	0.688	3.668
C	−2.269	−1.923	2.639
H	−2.259	1.700	4.056
H	−2.261	−2.931	2.244
C	−1.099	0.130	3.150
C	−1.106	−1.160	2.628
H	−0.184	0.709	3.144
H	−0.196	−1.576	2.213
C	−5.502	1.450	3.341
H	−6.466	1.653	3.816
H	−4.902	2.353	3.477
C	−7.740	−4.792	1.452
H	−6.778	−4.994	0.974
H	−8.340	−5.695	1.316

IF hexamer:

C	−4.233	0.386	−1.998
C	−4.796	−0.785	−2.499
C	−5.719	−1.495	−1.746
C	−4.587	0.867	−0.741
C	−5.517	0.173	0.008
C	−6.076	−1.007	−0.497
C	−6.176	0.542	1.339
C	−7.236	−0.545	1.475
C	−7.090	−1.470	0.432
C	−7.885	−2.595	0.370
C	−8.880	−2.715	1.305
C	−8.320	−0.608	2.332
C	−9.184	−1.693	2.211
H	−3.526	0.939	−2.601
H	−4.510	−1.139	−3.481
H	−6.169	−2.400	−2.137
H	−4.147	1.786	−0.374
H	−7.724	−3.349	−0.390
H	−8.499	0.162	3.073
C	−10.890	−3.278	2.359
C	−10.472	−2.018	2.829
C	−11.299	−1.279	3.663
C	−12.546	−1.791	4.004
H	−13.206	−1.215	4.640
C	−12.962	−3.027	3.525
H	−13.943	−3.403	3.784
C	−12.127	−3.781	2.702
H	−12.462	−4.737	2.320
H	−10.987	−0.311	4.034
C	−9.842	−3.860	1.415
C	−10.432	−4.081	−0.024
H	−10.502	−2.010	7.152
H	−11.592	−4.129	6.529
C	−10.061	−2.632	6.384
C	−10.679	−3.831	6.033
C	−8.890	−2.241	5.759
C	−10.137	−4.651	5.050
H	−8.401	−1.314	6.032
H	−10.632	−5.578	4.791
C	−8.355	−3.065	4.781
C	−8.970	−4.262	4.423
H	−4.114	−0.141	5.098
C	−7.173	−2.896	3.969
C	−8.164	−4.972	3.341
H	−6.414	−1.049	4.724
C	−6.293	−1.846	4.002
C	−7.030	−3.959	3.057
H	−0.536	−4.954	1.243
C	−5.279	−1.834	3.090
C	−6.007	−3.927	2.115
C	−4.279	−0.732	2.969
C	−5.125	−2.833	2.123
H	−5.898	−4.715	1.378
C	−3.460	−1.216	1.778
C	−3.989	−2.429	1.281
C	−2.405	−0.596	1.144
C	−3.438	−3.001	0.143
H	−2.007	0.340	1.518
H	−3.840	−3.921	−0.261
C	−1.854	−1.179	0.004
C	−2.374	−2.368	−0.492
H	−1.034	−0.693	−0.508
H	−1.950	−2.807	−1.386
C	−5.105	0.601	2.558
H	−5.648	0.936	3.445
H	−4.389	1.386	2.307
C	−9.084	−5.164	2.033
H	−8.458	−5.577	1.237
H	−9.849	−5.910	2.257
H	−8.987	−1.630	−2.574
H	−11.288	−3.260	−1.813
C	−11.105	−2.848	−0.816
H	−7.748	0.162	−4.184
H	−13.728	−4.063	−0.544
C	−9.365	−1.029	−1.755
H	−6.309	1.959	−5.097
C	−7.431	0.982	−3.551
C	−13.622	−3.068	−0.127
C	−10.373	−1.493	−0.918
C	−12.415	−2.401	−0.189

C	−6.631	1.999	−4.063
C	−8.791	0.220	−1.478
C	−7.842	1.049	−2.226
C	−14.717	−2.441	0.472
H	−15.672	−2.949	0.511
C	−10.965	−0.578	−0.019
C	−12.286	−1.104	0.340
C	−6.239	3.067	−3.262
C	−9.104	0.906	−0.285
C	−7.438	2.125	−1.413
H	−5.623	3.854	−3.678
C	−14.584	−1.169	1.017
C	−10.290	0.586	0.364
C	−13.368	−0.493	0.957
C	−6.640	3.129	−1.926
C	−8.061	2.012	−0.029
H	−15.437	−0.695	1.488
H	−10.665	1.190	1.182
H	−13.277	0.503	1.371
H	−6.329	3.962	−1.305
H	−9.634	−4.471	−0.659
C	−6.985	1.878	1.157
H	−6.288	2.707	1.008
H	−7.512	2.080	2.093
H	−11.206	−4.852	0.007
H	−8.574	2.950	0.207
H	−9.086	−4.162	9.782
H	−9.999	−6.276	8.904
C	−8.661	−4.600	8.887
C	−9.180	−5.789	8.391
C	−7.600	−3.965	8.251
C	−8.629	−6.372	7.251
H	−7.198	−3.044	8.656
H	−9.026	−7.309	6.877
C	−7.048	−4.536	7.113
C	−7.575	−5.750	6.617
H	−2.604	0.045	4.219
C	−5.912	−4.130	6.271
C	−6.756	−6.232	5.424
H	−5.139	−2.248	7.017
C	−5.031	−3.036	6.279
C	−5.758	−5.128	5.304
H	0.557	−2.838	1.868
C	−4.007	−3.003	5.337
C	−4.745	−5.116	4.392
C	−2.872	−1.990	5.054
C	−3.865	−4.065	4.425
H	−4.623	−5.912	3.669
C	−2.067	−2.701	3.973
C	−2.683	−3.897	3.614
C	−0.898	−2.314	3.346
C	−2.148	−4.722	2.636
H	−0.402	−1.388	3.606
H	−2.639	−5.648	2.362
C	−0.357	−3.134	2.363
C	−0.976	−4.332	2.011
C	−3.454	−0.624	4.373
C	−7.582	−6.339	4.020
H	−8.431	−7.009	4.174
H	−6.922	−6.821	3.295
H	2.174	−5.743	3.752
H	−6.518	−5.828	11.874
H	−7.501	−7.906	10.993
C	−6.232	−6.181	10.892
C	−6.795	−7.352	10.390
C	−5.311	−5.469	10.139
C	−6.442	−7.831	9.132
H	−4.861	−4.565	10.530
H	−6.883	−8.750	8.765
C	−4.954	−5.956	8.889
C	−5.514	−7.135	8.383
C	−3.942	−5.491	7.960
C	−4.855	−7.503	7.051
H	−3.307	−3.615	8.784
C	−3.147	−4.367	8.023
C	−3.797	−6.415	6.914
H	2.912	−3.556	4.612
C	−2.153	−4.245	7.087
C	−2.714	−6.351	6.057
C	−1.192	−3.100	6.978
C	−1.850	−5.266	6.179
H	−2.535	−7.119	5.315
C	−0.143	−3.681	6.034
C	−0.562	−4.940	5.562
C	1.094	−3.179	5.692
C	0.265	−5.679	4.728
H	1.430	−2.223	6.076
H	−0.047	−6.646	4.355
C	1.930	−3.931	4.869
C	1.513	−5.167	4.388
C	−1.951	−1.797	6.361
C	−5.928	−7.563	5.834
H	−1.187	−1.050	6.136
H	−2.578	−1.385	7.158
H	−6.643	−8.349	6.086
H	−5.386	−7.898	4.946
H	4.407	−6.258	6.902
H	−4.715	−8.929	13.485
H	−5.399	−10.822	12.064
C	−4.394	−8.967	12.451

C	−4.785	−10.034	11.648
C	−3.595	−7.948	11.940
C	−4.385	−10.094	10.312
H	−3.279	−7.128	12.574
H	−4.695	−10.926	9.690
C	−3.186	−8.012	10.614
C	−3.589	−9.087	9.800
C	−2.238	−7.181	9.867
C	−2.968	−8.972	8.416
H	−2.041	−5.333	10.966
C	−1.665	−5.932	10.145
C	−1.925	−7.865	8.672
H	4.641	−4.006	7.883
C	−0.658	−5.466	9.309
C	−0.740	−7.543	8.023
C	0.074	−4.111	9.208
C	−0.065	−6.380	8.408
H	−0.365	−8.146	7.204
C	1.384	−4.556	8.581
C	1.255	−5.852	8.049
C	2.591	−3.889	8.521
C	2.338	−6.462	7.431
H	2.696	−2.895	8.940
H	2.248	−7.458	7.016
C	3.686	−4.514	7.921
C	3.554	−5.785	7.373
C	−4.045	−8.838	7.231
H	−3.519	−9.039	6.295
H	−4.741	−9.668	7.380
C	−0.601	−2.879	8.417
H	−1.399	−2.490	9.052
H	0.172	−2.107	8.386
H	0.256	−3.699	10.205
H	−2.453	−9.908	8.178

IF decamer:

H	−11.032	−4.198	6.405	C	−6.295	−5.417	7.231
H	−11.220	−6.529	5.565	C	−6.566	−6.698	6.782
C	−10.214	−4.638	5.849	H	−2.471	−0.285	5.602
C	−10.321	−5.959	5.372	C	−5.045	−4.987	6.644
C	−9.054	−3.886	5.604	C	−5.479	−7.197	5.857
C	−9.266	−6.541	4.649	H	−4.676	−3.035	7.512
H	−8.973	−2.866	5.956	C	−4.341	−3.794	6.818
H	−9.345	−7.556	4.282	C	−4.595	−5.987	5.788
C	−8.021	−4.482	4.880	H	1.144	−1.991	2.719
C	−8.117	−5.783	4.412	C	−3.192	−3.620	6.031
H	−3.847	−0.987	6.484	C	−3.476	−5.791	4.963
C	−6.765	−3.914	4.433	C	−2.260	−2.445	6.022
C	−6.880	−6.199	3.658	C	−2.811	−4.570	5.088
H	−6.602	−2.002	5.470	H	−3.154	−6.540	4.253
C	−6.169	−2.672	4.738	C	−1.367	−2.804	4.855
C	−6.163	−4.845	3.585	C	−1.699	−4.040	4.330
H	0.524	−4.206	1.780	C	−0.334	−2.047	4.290
C	−4.972	−2.349	4.074	C	−1.038	−4.572	3.222
C	−5.057	−4.457	2.808	H	−0.072	−1.079	4.695
C	−4.028	−1.160	4.284	H	−1.317	−5.534	2.813
C	−4.513	−3.177	3.048	C	0.347	−2.563	3.174
H	−4.629	−5.123	2.071	C	−0.005	−3.819	2.641
C	−3.183	−1.293	3.044	C	−3.146	−1.166	5.642
C	−3.448	−2.472	2.364	C	−6.081	−7.403	4.388
C	−2.234	−0.390	2.556	H	−6.762	−8.280	4.420
C	−2.783	−2.803	1.184	H	−5.239	−7.684	3.721
H	−2.027	0.533	3.082	H	2.271	−5.823	3.894
H	−3.005	−3.721	0.656	H	−7.187	−5.852	11.491
C	−1.557	−0.701	1.365	H	−7.823	−8.136	10.746
C	−1.828	−1.904	0.684	C	−6.556	−6.386	10.792
H	−0.819	−0.014	0.971	C	−6.917	−7.680	10.370
H	−1.300	−2.134	−0.232	C	−5.381	−5.784	10.312
H	−9.057	−4.863	9.111	C	−6.108	−8.384	9.460
H	−9.552	−7.169	8.327	H	−5.101	−4.789	10.630
C	−8.357	−5.374	8.464	H	−6.387	−9.375	9.129
C	−8.638	−6.680	8.019	C	−4.593	−6.502	9.411
C	−7.173	−4.729	8.070	C	−4.943	−7.772	8.985
C	−7.739	−7.353	7.173	C	−3.366	−6.122	8.748
H	−6.955	−3.723	8.402	C	−3.944	−8.316	7.990
H	−7.956	−8.353	6.823	H	−2.906	−4.151	9.535

C	−2.619	−4.944	8.858
C	−2.994	−7.155	7.890
H	2.962	−3.578	4.706
C	−1.492	−4.830	8.029
C	−1.877	−7.034	7.049
C	−0.534	−3.673	7.938
C	−1.153	−5.839	7.130
H	−1.601	−7.819	6.358
C	0.363	−4.145	6.817
C	−0.010	−5.398	6.362
C	1.441	−3.470	6.233
C	0.657	−6.029	5.310
H	1.735	−2.490	6.584
H	0.349	−7.004	4.958
C	2.128	−4.086	5.173
C	1.737	−5.359	4.712
C	−1.386	−2.371	7.418
C	−4.684	−8.473	6.534
H	−0.675	−1.524	7.318
H	−2.092	−2.101	8.231
H	−5.410	−9.309	6.614
H	−3.906	−8.795	5.810
H	3.988	−7.244	6.016
H	−5.392	−6.962	13.731
H	−6.097	−9.233	13.010
C	−4.802	−7.503	13.003
C	−5.202	−8.791	12.594
C	−3.642	−6.917	12.471
C	−4.446	−9.504	11.647
H	−3.331	−5.928	12.779
H	−4.753	−10.491	11.329
C	−2.907	−7.645	11.533
C	−3.294	−8.909	11.122
C	−1.699	−7.285	10.824
C	−2.344	−9.474	10.091
H	−1.195	−5.307	11.571
C	−0.933	−6.114	10.900
C	−1.367	−8.333	9.967
H	4.717	−4.989	6.766
C	0.184	−6.031	10.054
C	−0.254	−8.247	9.117
C	1.160	−4.890	9.929
C	0.500	−7.068	9.178
H	0.002	−9.050	8.438
C	2.060	−5.418	8.836
C	1.664	−6.677	8.414
C	3.166	−4.791	8.253
C	2.338	−7.360	7.400
H	3.479	−3.807	8.579
H	2.015	−8.340	7.077
C	3.860	−5.460	7.231
C	3.447	−6.738	6.805
C	−3.153	−9.614	8.653
H	−2.414	−9.964	7.900
H	−3.899	−10.428	8.770
C	0.339	−3.576	9.344
H	−0.363	−3.252	10.141
H	1.076	−2.755	9.215
H	10.969	−12.087	14.699
H	9.174	−10.978	12.458
H	7.114	−12.012	13.410
H	7.424	−9.799	10.284
H	12.839	−13.075	17.079
H	8.884	−13.151	15.559
H	10.738	−14.216	17.787
H	5.389	−10.830	11.291
C	10.338	−11.553	15.397
H	7.630	−16.954	19.703
H	0.790	−12.328	12.033
C	8.584	−10.437	13.186
C	7.424	−11.023	13.718
H	2.452	−13.512	14.060
C	9.164	−12.156	15.877
H	5.875	−15.840	17.956
H	4.145	−14.689	16.046
C	6.859	−9.260	11.033
H	8.393	−17.725	21.984
C	12.139	−12.565	17.726
H	4.977	−12.633	14.617
H	−0.670	−12.767	12.954
H	6.254	−17.657	20.584
C	10.955	−13.210	18.119
C	5.714	−9.844	11.597
H	12.756	−15.073	20.233
H	8.459	−14.905	18.676
C	6.929	−16.775	20.545
C	0.085	−11.966	12.811
H	10.385	−15.938	20.719
H	3.295	−11.455	12.567
H	1.636	−10.260	10.507
H	3.692	−9.608	9.184
H	6.688	−13.790	16.653
H	1.001	−13.968	14.988
H	11.605	−9.803	15.445
H	14.814	−13.739	19.785
C	1.751	−13.162	14.847
C	10.698	−10.259	15.820
H	4.458	−16.418	18.869
C	5.169	−15.570	18.770
H	5.811	−18.476	23.104

C	3.443	−14.366	16.844
H	2.706	−15.187	16.972
C	7.811	−16.782	21.903
C	8.984	−9.149	13.595
C	−0.709	−10.651	12.171
H	−3.090	−11.661	13.467
H	5.700	−8.558	8.143
H	9.879	−8.707	13.180
C	12.836	−14.053	20.585
C	4.715	−11.826	15.288
C	8.123	−14.146	19.370
C	7.277	−7.976	11.437
C	9.951	−15.269	21.450
C	6.689	−10.296	14.656
C	4.021	−8.625	9.494
C	3.045	−10.643	13.237
C	13.996	−13.300	20.341
C	12.419	−11.258	18.172
C	1.388	−9.449	11.179
H	8.161	−7.535	10.996
C	0.947	−11.845	14.228
C	6.042	−15.497	20.166
C	8.375	−11.438	16.778
C	6.401	−12.997	17.331
H	13.334	−10.770	17.864
C	2.622	−13.052	16.259
C	10.077	−12.522	18.958
C	5.151	−8.029	8.910
C	5.010	−9.119	12.560
C	6.017	−17.554	23.631
C	8.754	−15.592	22.114
C	4.317	−14.268	18.250
C	−2.771	−10.676	13.780
C	5.481	−10.656	15.365
C	0.277	−9.520	12.034
C	6.965	−16.650	23.143
C	3.598	−11.911	16.134
C	−1.632	−10.084	13.224
C	8.827	−12.954	19.545
C	6.974	−14.321	20.157
C	1.933	−10.713	14.091
C	3.818	−9.475	13.297
C	7.148	−11.819	17.441
C	9.889	−9.556	16.730
H	−1.405	−12.875	15.560
C	11.802	−13.458	21.310
C	8.228	−8.436	14.542
C	5.274	−13.112	18.159
C	10.547	−14.026	21.756
H	3.855	−16.864	21.491
C	3.336	−7.913	10.481
C	2.157	−8.279	11.235
C	7.076	−9.031	15.067
C	8.725	−10.168	17.205
C	6.552	−7.265	12.409
H	2.047	−15.453	19.603
C	5.339	−17.243	24.821
C	14.102	−11.980	20.819
H	4.602	−17.931	25.215
C	11.520	−10.586	19.017
C	5.413	−7.857	12.965
C	0.040	−11.288	15.300
C	10.347	−11.242	19.407
C	5.149	−15.139	21.332
H	0.304	−14.135	17.609
C	−1.084	−11.891	15.874
C	−3.495	−9.965	14.752
C	5.572	−6.748	9.319
H	−4.379	−10.406	15.193
C	4.117	−15.896	21.897
C	1.722	−12.524	17.352
H	15.001	−11.409	20.626
C	3.419	−13.798	19.371
C	8.295	−14.765	23.140
C	7.230	−15.471	23.823
H	10.168	−8.564	17.061
C	−0.036	−8.466	12.891
C	5.149	−9.608	16.222
C	2.342	−14.473	19.954
H	6.444	−6.298	8.863
C	−1.228	−8.822	13.628
C	0.616	−13.152	17.935
C	3.281	−10.873	17.010
H	8.535	−7.449	14.861
C	6.593	−13.372	21.100
C	8.376	−11.954	20.401
C	3.504	−8.421	14.155
C	1.624	−9.662	14.954
C	11.897	−12.157	21.778
C	6.776	−10.786	18.299
C	3.742	−6.653	10.888
H	6.872	−6.280	12.722
C	9.944	−13.096	22.604
C	4.935	−12.103	19.058
C	13.047	−11.398	21.542
C	6.125	−8.467	16.098
C	1.849	−7.228	12.098
H	11.737	−9.586	19.367
C	4.866	−6.051	10.314
C	5.609	−16.041	25.503

C	5.481	−13.903	21.858
C	0.446	−10.028	15.708
C	7.725	−9.624	18.199
C	4.035	−9.694	17.072
C	2.118	−11.265	17.774
C	4.491	−7.290	14.018
C	9.259	−10.744	20.332
C	8.838	−13.485	23.381
C	−1.790	−11.194	16.870
C	6.564	−15.140	25.004
C	−3.077	−8.681	15.156
C	7.257	−12.151	21.225
C	3.792	−12.544	19.826
C	0.737	−7.298	12.951
C	3.435	−15.381	23.013
C	2.393	−8.492	15.009
C	5.658	−10.908	19.140
C	−1.932	−8.097	14.592
H	−2.662	−11.644	17.325
C	10.660	−11.742	22.532
C	1.654	−13.857	21.014
H	2.638	−15.954	23.467
C	2.834	−6.096	11.960
C	6.933	−8.326	17.537
C	5.334	−7.159	15.444
C	−0.079	−12.483	18.957
C	8.464	−9.468	19.656
H	0.820	−14.365	21.481
H	5.081	−15.811	26.419
H	13.125	−10.383	21.909
C	9.861	−10.538	21.802
C	3.696	−5.975	13.378
H	7.679	−7.512	17.420
H	5.187	−5.066	10.629
C	4.819	−13.371	22.966
H	6.088	−6.356	15.308
C	−0.239	−9.317	16.695
H	−3.642	−8.142	15.905
H	−0.935	−12.954	19.422
H	9.190	−8.632	19.576

C	−1.369	−9.912	17.278
C	3.786	−14.125	23.546
H	3.779	−8.892	17.751
H	6.786	−14.223	25.532
C	1.444	−10.583	18.788
H	8.409	−12.819	24.118
C	3.125	−11.914	20.879
H	6.934	−11.402	21.936
H	0.487	−6.486	13.621
H	2.145	−7.681	15.681
H	−1.607	−7.111	14.897
C	2.045	−12.585	21.476
H	4.452	−5.175	13.234
H	10.968	−11.440	23.556
H	10.542	−9.661	21.770
H	5.382	−10.123	19.831
C	0.334	−11.205	19.383
H	6.195	−7.977	18.289
H	4.617	−6.797	16.212
H	7.686	−9.146	20.380
H	9.019	−10.257	22.469
H	2.991	−5.613	14.155
H	5.098	−12.409	23.375
H	0.090	−8.334	17.005
H	1.767	−9.602	19.112
H	−1.919	−9.384	18.046
H	3.257	−13.739	24.407
H	3.432	−10.939	21.231
H	1.511	−12.121	22.294
H	−0.207	−10.699	20.172
C	−1.553	−10.782	10.745
C	2.031	−4.780	11.341
H	1.330	−4.429	12.127
H	2.781	−3.973	11.199
H	−2.307	−11.585	10.881
H	−0.836	−11.144	9.977
H	−7.188	−6.501	2.634
H	−4.609	−0.217	4.203

References

(1) Neese, F. The ORCA program system. *Wiley Interdiscip. Rev.: Comput. Mol. Sci.* **2012**, *2*, 73–78.

(2) Neese, F. Software update: the ORCA program system, version 4.0. *Wiley Interdiscip. Rev.: Comput. Mol. Sci.* **2018**, *8*, e1327.

(3) Becke, A. D. A new mixing of Hartree–Fock and local density-functional theories. *J. Chem. Phys.* **1993**, *98*, 1372–1377.

(4) Becke, A. D. Density-functional exchange-energy approximation with correct asymptotic behavior. *Phys. Rev. A* **1988**, *38*, 3098–3100.

(5) Lee, C.; Yang, W.; Parr, R. G. Development of the Colle-Salvetti correlation-energy formula into a functional of the electron density. *Phys. Rev. B* **1988**, *37*, 785–789.

(6) Schäfer, A.; Huber, C.; Ahlrichs, R. Fully optimized contracted Gaussian basis sets of triple zeta valence quality for atoms Li to Kr. *J. Chem. Phys.* **1994**, *100*, 5829–5835.

(7) Izsák, R.; Neese, F. An overlap fitted chain of spheres exchange method. *J. Chem. Phys.* **2011**, *135*, 144105.

(8) Neese, F.; Wennmohs, F.; Hansen, A.; Becker, U. Efficient, approximate and parallel Hartree–Fock and hybrid DFT calculations. A 'chain-of-spheres'algorithm for the Hartree–Fock exchange. *Chem. Phys.* **2009**, *356*, 98–109.

(9) Weigend, F. Accurate Coulomb-fitting basis sets for H to Rn. *Phys. Chem. Chem. Phys.* **2006**, *8*, 1057–1065.

(10) Grimme, S.; Antony, J.; Ehrlich, S.; Krieg, H. A consistent and accurate ab initio parametrization of density functional dispersion correction (DFT-D) for the 94 elements H-Pu. *J. Chem. Phys.* **2010**, *132*, 154104.

(11) Becke, A. D.; Johnson, E. R. A density-functional model of the dispersion interaction. *J. Chem. Phys.* **2005**, *123*, 154101.

(12) Grimme, S.; Ehrlich, S.; Goerigk, L. Effect of the damping function in dispersion corrected density functional theory. *J. Comput. Chem.* **2011**, *32*, 1456–1465.

(13) Sirianni, D. A.; Alenaizan, A.; Cheney, D. L.; Sherrill, C. D. Assessment of density functional methods for geometry optimization of bimolecular van der Waals complexes. *J. Chem. Theory Comput.* **2018**, *14*, 3004–3013.

(14) Sedlak, R.; Janowski, T.; Pitonak, M.; Rezac, J.; Pulay, P.; Hobza, P. Accuracy of quantum chemical methods for large noncovalent complexes. *J. Chem. Theory Comput.* **2013**, *9*, 3364–3374.

(15) Silva, N. J.; Machado, F. B.; Lischka, H.; Aquino, A. J. π–π stacking between polyaromatic hydrocarbon sheets beyond dispersion interactions. *Phys. Chem. Chem. Phys.* **2016**, *18*, 22300–22310.

(16) Hanwell, M. D.; Curtis, D. E.; Lonie, D. C.; Vandermeersch, T.; Zurek, E.; Hutchison, G. R. Avogadro: an advanced semantic chemical editor, visualization, and analysis platform. *J. Cheminformatics* **2012**, *4*, 1–17.

(17) Te Velde, G. t.; Bickelhaupt, F. M.; Baerends, E. J.; Fonseca Guerra, C.; van Gisbergen, S. J.; Snijders, J. G.; Ziegler, T. Chemistry with ADF. *J. Comput. Chem.* **2001**, *22*, 931–967.

(18) Hirata, S.; Head-Gordon, M. Time-dependent density functional theory within the Tamm–Dancoff approximation. *Chem. Phys. Lett.* **1999**, *314*, 291–299.

(19) Zhao, Y.; Truhlar, D. G. The M06 suite of density functionals for main group thermochemistry, thermochemical kinetics, noncovalent interactions, excited states, and transition elements: two new functionals and systematic testing of four M06-class functionals and 12 other functionals. *Theor. Chem. Acc.* **2008**, *120*, 215–241.

(20) Zhao, Y.; Truhlar, D. G. A new local density functional for main-group thermochemistry, transition metal bonding, thermochemical kinetics, and noncovalent interactions. *J. Chem. Phys.* **2006**, *125*, 194101.

(21) Van Lenthe, E.; Baerends, E. J. Optimized Slater-type basis sets for the elements 1–118. *J. Comput. Chem.* **2003**, *24*, 1142–1156.

(22) Zhao, Y.; Truhlar, D. G. Density functionals with broad applicability in chemistry. *Acc. Chem. Res.* **2008**, *41*, 157–167.

(23) Weigend, F.; Ahlrichs, R. Balanced basis sets of split valence, triple zeta valence and quadruple zeta valence quality for H to Rn: design and assessment of accuracy. *Phys. Chem. Chem. Phys.* **2005**, *7*, 3297–3305.

(24) Schäfer, A.; Horn, H.; Ahlrichs, R. Fully optimized contracted Gaussian basis sets for atoms Li to Kr. *J. Chem. Phys.* **1992**, *97*, 2571–2577.

(25) Bérces, A.; Dickson, R. M.; Fan, L.; Jacobsen, H.; Swerhone, D.; Ziegler, T. An implementation of the coupled perturbed Kohn-Sham equations: perturbation due to nuclear displacements. *Comput. Phys. Commun.* **1997**, *100*, 247–262.

(26) Wolff, S. K. Analytical second derivatives in the Amsterdam density functional package. *Int. J. Qantum Chem.* **2005**, *104*, 645–659.

(27) Seldenthuis, J. S.; Van Der Zant, H. S.; Ratner, M. A.; Thijssen, J. M. Vibrational excitations in weakly coupled single-molecule junctions: a computational analysis. *ACS Nano* **2008**, *2*, 1445–1451.

(28) Ruhoff, P. T.; Ratner, M. A. Algorithms for computing Franck–Condon overlap integrals. *Int. J. Quantum Chem.* **2000**, *77*, 383–392.

(29) May, V.; Kühn, O. *Charge and energy transfer dynamics in molecular systems*; John Wiley & Sons, 2000.

(30) Chai, J.-D.; Head-Gordon, M. Systematic optimization of long-range corrected hybrid density functionals. *J. Chem. Phys.* **2008**, *128*, 084106.

(31) Bokarev, S. I.; Bokareva, O. S.; Kühn, O. Electronic excitation spectrum of the photosensitizer $[Ir(ppy)_2(bpy)]^+$. *J. Chem. Phys.* **2012**, *136*, 214305.

(32) Plasser, F.; Dreuw, A. High-level ab initio computations of the absorption spectra of organic iridium complexes. *J. Phys. Chem. A* **2015**, *119*, 1023–1036.

(33) Atkins, A. J.; Talotta, F.; Freitag, L.; Boggio-Pasqua, M.; Gonzalez, L. Assessing excited state energy gaps with time-dependent density functional theory on Ru (II) complexes. *J. Chem. Theory Comput.* **2017**, *13*, 4123–4145.

(34) Sears, J. S.; Koerzdoerfer, T.; Zhang, C.-R.; Brédas, J.-L. Communication: Orbital instabilities and triplet states from time-dependent density functional theory and long-range corrected functionals. *J. Chem. Phys.* **2011**, *135*, 151103.

(35) Peach, M. J.; Tozer, D. J. Overcoming low orbital overlap and triplet instability problems in TDDFT. *J. Phys. Chem. A* **2012**, *116*, 9783–9789.

(36) Hartzler, D. A.; Slipchenko, L. V.; Savikhin, S. Triplet-triplet coupling in chromophore dimers: theory and experiment. *J. Phys. Chem. A* **2018**, *122*, 6713–6723.

(37) Schieschke, N.; Bold, B. M.; Dohmen, P. M.; Wehl, D.; Hoffmann, M.; Dreuw, A.; Elstner, M.; Höfener, S. Geometry dependence of excitonic couplings and the consequences for configuration-space sampling. *J. Comput. Chem.* **2021**, *42*, 1402–1418.

(38) Andrae, D.; Haeussermann, U.; Dolg, M.; Stoll, H.; Preuss, H. Energy-adjusted *ab-initio* pseudopotentials for the second and third row transition elements. *Theor. Chim. Acta* **1990**, *77*, 123–141.

(39) Case, D. A.; Aktulga, H. M.; Belfon, K.; Ben-Shalom, I.; Brozell, S. R.; Cerutti, D. S.; Cheatham III, T. E.; Cruzeiro, V. W. D.; Darden, T. A.; Duke, R. E., et al. *Amber 2021*; University of California, San Francisco, 2021.

(40) Jakalian, A.; Bush, B. L.; Jack, D. B.; Bayly, C. I. Fast, efficient generation of high-quality atomic charges. AM1-BCC model: I. Method. *J. Comput. Chem.* **2000**, *21*, 132–146.

(41) Jakalian, A.; Jack, D. B.; Bayly, C. I. Fast, efficient generation of high-quality atomic charges. AM1-BCC model: II. Parameterization and validation. *J. Comput. Chem.* **2002**, *23*, 1623–1641.

(42) Wang, J.; Wolf, R. M.; Caldwell, J. W.; Kollman, P. A.; Case, D. A. Development and testing of a general amber force field. *J. Comput. Chem.* **2004**, *25*, 1157–1174.

(43) Pastor, R. W.; Brooks, B. R.; Szabo, A. An analysis of the accuracy of Langevin and molecular dynamics algorithms. *Mol. Phys.* **1988**, *65*, 1409–1419.

(44) Loncharich, R. J.; Brooks, B. R.; Pastor, R. W. Langevin dynamics of peptides: The frictional dependence of isomerization rates of N-acetylalanyl-N'-methylamide. *Biopolymers* **1992**, *32*, 523–535.

(45) Izaguirre, J. A.; Catarello, D. P.; Wozniak, J. M.; Skeel, R. D. Langevin stabilization of molecular dynamics. *J. Chem. Phys.* **2001**, *114*, 2090–2098.

(46) Shao, Y.; Gan, Z.; Epifanovsky, E.; Gilbert, A. T.; Wormit, M.; Kussmann, J.; Lange, A. W.; Behn, A.; Deng, J.; Feng, X., et al. Advances in molecular quantum chemistry contained in the Q-Chem 4 program package. *Mol. Phys.* **2015**, *113*, 184–215.

(47) Hariharan, P.; Pople, J. Accuracy of AH_n equilibrium geometries by single determinant molecular orbital theory. *Mol. Phys.* **1974**, *27*, 209–214.

(48) Bussi, G.; Parrinello, M. Accurate sampling using Langevin dynamics. *Phys. Rev. E* **2007**, *75*, 056707.

(49) Martin, R. L. Natural transition orbitals. *J. Chem. Phys.* **2003**, *118*, 4775–4777.

(50) Nooijen, M.; Bartlett, R. J. A new method for excited states: similarity transformed equation-of-motion coupled-cluster theory. *J. Chem. Phys.* **1997**, *106*, 6441–6448.

(51) Nooijen, M.; Bartlett, R. J. Similarity transformed equation-of-motion coupled-cluster theory: details, examples, and comparisons. *J. Chem. Phys.* **1997**, *107*, 6812–6830.

(52) Dutta, A. K.; Nooijen, M.; Neese, F.; Izsák, R. Automatic active space selection for the similarity transformed equations of motion coupled cluster method. *J. Chem. Phys.* **2017**, *146*, 074103.

(53) Dutta, A. K.; Nooijen, M.; Neese, F.; Izsák, R. Exploring the accuracy of a low scaling similarity transformed equation of motion method for vertical excitation energies. *J. Chem. Theory Comput.* **2018**, *14*, 72–91.

(54) Sous, J.; Goel, P.; Nooijen, M. Similarity transformed equation of motion coupled cluster theory revisited: a benchmark study of valence excited states. *Mol. Phys.* **2014**, *112*, 616–638.

(55) Dutta, A. K.; Neese, F.; Izsák, R. Towards a pair natural orbital coupled cluster method for excited states. *J. Chem. Phys.* **2016**, *145*, 034102.

(56) Head-Gordon, M.; Rico, R. J.; Oumi, M.; Lee, T. J. A doubles correction to electronic excited states from configuration interaction in the space of single substitutions. *Chem. Phys. Lett.* **1994**, *219*, 21–29.

(57) Harbach, P. H.; Wormit, M.; Dreuw, A. The third-order algebraic diagrammatic construction method (ADC (3)) for the polarization propagator for closed-shell molecules: efficient implementation and benchmarking. *J. Chem. Phys.* **2014**, *141*, 064113.

(58) Wormit, M.; Rehn, D. R.; Harbach, P. H.; Wenzel, J.; Krauter, C. M.; Epifanovsky, E.; Dreuw, A. Investigating excited electronic states using the algebraic diagrammatic construction (ADC) approach of the polarisation propagator. *Mol. Phys.* **2014**, *112*, 774–784.

(59) Thouless, D. J. Electrons in disordered systems and the theory of localization. *Phys. Rep.* **1974**, *13*, 93–142.

(60) Jimenez, R.; Dikshit, S. N.; Bradforth, S. E.; Fleming, G. R. Electronic excitation transfer in the LH2 complex of Rhodobacter sphaeroides. *J. Phys. Chem.* **1996**, *100*, 6825–6834.

(61) Meier, T.; Chernyak, V.; Mukamel, S. Multiple exciton coherence sizes in photosynthetic antenna complexes viewed by pump- probe spectroscopy. *J. Phys. Chem. B* **1997**, *101*, 7332–7342.

(62) Novoderezhkin, V.; Monshouwer, R.; Van Grondelle, R. Exciton (de) localization in the LH2 antenna of Rhodobacter sphaeroides as revealed by relative difference absorption measurements of the LH2 antenna and the B820 subunit. *J. Phys. Chem. B* **1999**, *103*, 10540–10548.

(63) Scholes, G. D. Limits of exciton delocalization in molecular aggregates. *Faraday Disc.* **2020**, *221*, 265–280.

(64) Giannini, S.; Peng, W.-T.; Cupellini, L.; Padula, D.; Carof, A.; Blumberger, J. Exciton transport in molecular organic semiconductors boosted by transient quantum delocalization. *Nat. Commun.* **2022**, *13*, 1–13.

(65) Hartnett, P. E.; Mauck, C. M.; Harris, M. A.; Young, R. M.; Wu, Y.-L.; Marks, T. J.; Wasielewski, M. R. Influence of anion delocalization on electron transfer in a covalent porphyrin donor–perylenediimide dimer acceptor system. *J. Am. Chem. Soc.* **2017**, *139*, 749–756.

(66) Kaufmann, C.; Bialas, D.; Stolte, M.; Würthner, F. Discrete π-stacks of perylene bisimide dyes within folda-dimers: insight into long-and short-range exciton coupling. *J. Am. Chem. Soc.* **2018**, *140*, 9986–9995.

(67) Lindquist, R. J.; Lefler, K. M.; Brown, K. E.; Dyar, S. M.; Margulies, E. A.; Young, R. M.;

Wasielewski, M. R. Energy flow dynamics within cofacial and slip-stacked perylene-3, 4-dicarboximide dimer models of π-aggregates. *J. Am. Chem. Soc.* **2014**, *136*, 14912–14923.

(68) Margulies, E. A.; Miller, C. E.; Wu, Y.; Ma, L.; Schatz, G. C.; Young, R. M.; Wasielewski, M. R. Enabling singlet fission by controlling intramolecular charge transfer in π-stacked covalent terrylenediimide dimers. *Nat. Chem.* **2016**, *8*, 1120–1125.

(69) Liu, K.; Mukhopadhyay, A.; Ashcraft, A.; Liu, C.; Levy, A.; Blackwelder, P.; Olivier, J.-H. Reconfiguration of π-conjugated superstructures enabled by redox-assisted assembly. *Chem. Commun.* **2019**, *55*, 5603–5606.

(70) Gao, F.; Zhao, Y.; Liang, W. Vibronic spectra of perylene bisimide oligomers: effects of intermolecular charge-transfer excitation and conformational flexibility. *J. Phys. Chem. B* **2011**, *115*, 2699–2708.

(71) Macedo, A. G.; Christopholi, L. P.; Gavim, A. E.; de Deus, J. F.; Teridi, M. A. M.; Yusoff, A.; bin Mohd, R.; da Silva, W. J. Perylene derivatives for solar cells and energy harvesting: a review of materials, challenges and advances. *J. Mater. Sci. Mater. Electron.* **2019**, *30*, 15803–15824.

(72) Canonne, P.; Belanger, D.; Lemay, G.; Foscolos, G. B. One-step spiroannulation. Synthesis of spiro γ- and δ-lactones. *J. Org. Chem.* **1981**, *46*, 3091–3097.

(73) Cao, J.; Silbey, R. J. Optimization of exciton trapping in energy transfer processes. *J. Phys. Chem. A* **2009**, *113*, 13825–13838.
