# Supplementary material for: Molecular Wires for Efficient Long-Distance Triplet Energy Transfer
Source: J Phys Chem Lett. 2022 Oct 10;13(41):9679–87. doi: 10.1021/acs.jpclett.2c02616 (PMC9589895; doi:10.1021/acs.jpclett.2c02616)
Supplement: Supplementary file 2 — jz2c02616_si_002.pdf [file jz2c02616_si_002.pdf]

jz-2022-02616m.R1

Name: Peer Review Information for "Molecular Wires for Efficient Long-Distance Triplet Energy Transfer"

First Round of Reviewer Comments

Reviewer: 1

Comments to the Author

The topic of this paper is original. This theoretical discussion of long range triplet transfer uses a combination of phenomenological theories and a variety of computational chemistry methods making the predictions quite robust.

The chemistry proposed in scheme 1 is quite ambitious from the synthetic point of view – probably the same effect can be achieved with a single linker and bulky functional group.

The chemistry proposed in Figure 4 could be wrong the “I” in PDI is for “imides” but there is no nitrogen in the molecule. Nobody replaces the C=O bond with the C-C bond in these molecules. This particular proposal should be reconsidered to maintain the credibility of the paper.

Reviewer: 2

Comments to the Author

The authors investigate triplet exciton transfer between molecular donor and acceptor connected by a molecular bridge comprised of pi-conjugated organic units. Starting from an experimentally investigated system, where TE transport was slow and in the incoherent hopping regime, they propose here a modification of the bridge based on established design principles. They then carry out electronic structure calculations to characerize triplet states, excitonic couplings, reorganization energies etc. This is used for parametrization of a Liouville equation for propagation of the triplet exciton across the D-B-A system. Fast triplet exciton transport along this bridge is predicted with effective intra-bridge TE rates in the order of 10 fs at 6 repeat units.

The work makes a theoretical prediction for a fast TE wire that awaits experimental verification. The work is thus not only of interest to theoreticians but also to synthetic and experimental physical chemists interested in singlet fission and triplet exciton transport. The paper is well motivated, certain parts of the manuscript could be improved and better explained. Overall, I recommend publication in JPCL after minor corrections.

p 4 l54: In my view the condition V_rms >= \lambda/2 is correct only if lambda is defined as
the reorganization energy for exciton transfer from donor to acceptor
accounting for reorganization energy of _both_ molecules, donor and acceptor
(lambda = lambda_D + lambda_A)
However, it appears in this paper lambda is defined to be the monomer reorganization energy
only (lambda_D or lambda_A)
This issue is important because V_rms in their designed molecule is 0.14 eV
and lambda_D + \lambda_A = 0.27 + 0.27 = 0.54 eV, so V_rms >= \lambda/2
does not seem to be fulfilled. Please clarify.

p10 l 30: Are the H_j,i elements on the Liouville Equation assumed to be time-independent?
If so, then some important nuclear dynamics effects are probably not captured in this equation,
e.g. diagonal and off-diagonal disorder, spontaneous TE localization, back reaction
from electronic to nuclear dynamcis, non-adiabatic transitions etc
The approximations of the Liouville equation used should be explained. (the authors do say that
the IPR does not change much during MD but the MD is probably just ground state
dynamics with classical force fields)

p 11 l 10: The authors should explain a bit better the physical meaning of \tau_trap and \tau_br
instead of referring to Ref 54.

Scheme 1: the bridge molecules proposed look rather strained - is there any
evidence that these molecules actually exist / could be synthesized?

p 13 l 55: TD-DFT (ωB97/def2-SVP), usually one would expect a basis set of at least
double zeta quality - were the results for SVP basis set checked against larger basis sets?

p 15 l17: 1/k_br = 10 fs
Could the authors show how this value depends on the bridge length?
How much faster is TE transport in this wire compared to the one measured
experimentally by Vura-Weis et al?

p 4 l 44 One important parameter is also the thermal fluctuations of V
(\sigma_V), which should be small in the coherent/delocalized regime.

p 3 l 38 Walls -> Waals

Author's Response to Peer Review Comments:

Please see attached pdf

Department of Physics
University of Cyprus
P. O. Box 20537
Nicosia 1678
Cyprus

Friday, September 30th, 2022

To:
Prof. Editor
Senior Editor: The Journal of Physical Chemistry Letters

Dear Prof. Editor

Below please find our responses to the referee comments regarding the submitted manuscript entitled *"Molecular wires for efficient long-distance triplet energy transfer."*

We have addressed all of the referee comments (shown in italics below, each comment followed by our response). We also made changes to the main text and to the Supporting Information (SI) in response to the comments (in the revised main text these changes are highlighted). We thank the referees for their input that helped us improve the presentation.

Reviewer: 1

Recommendation: This paper is publishable subject to minor revisions noted. Further review is not needed.

Comments:

The chemistry proposed in scheme 1 is quite ambitious from the synthetic point of view – probably the same effect can be achieved with a single linker and bulky functional group.
The chemistry proposed in Figure 4 could be wrong the "I" in PDI is for "imides" but there is no nitrogen in the molecule. Nobody replaces the C=O bond with the C-C bond in these molecules. This particular proposal should be reconsidered to maintain the credibility of the paper.

Author’s answer:

We fully understand that the paper is ambitious from a synthetic point of view. We did try computations on several standard functional groups (i.e., already synthesized), some of which were presented in the original SI section 6 (see Figure S6). None of them had all of the required properties, i.e., maximum intermonomer distance at most van der Waals (VdW), low dynamic disorder (low distance fluctuations, low slippage and torsional motions). Following the referee’s comments, we tried more structures for the revised manuscript (some are included in the new SI in section 6 in Figure S7, and one in the main text in Figure 4(d)). The only one that does the job among the structures we tried is also synthetically challenging. It is included in the main text as described below. Importantly we did not find any single-linker polymeric structure that does not twist in the molecular dynamics simulations so as to break the pi-stacking.

With respect to the mislabeled molecule of Fig. **4**, we have corrected the nomenclature (the PDI systems that were originally considered were described in the original SI section 6). With respect to the replacement of the C=O bond with the C-C bond in the system of Figure 4(a-c), we had found an article that does the replacement in a related system but did not cite the article. We now cite the in the revised manuscript so as to retain credibility with the synthetic community article (Canonne, P.; Belanger, D.; Lemay, G.; Foscolos, G. B. One-step spiroannulation. Synthesis of spiro γ- and δ-lactones, J. Org. Chem. **1981**, *46*, 3091-3097). In addition to this system, we have also added in Figure 4 a dicyclopenta[ghi,pqr]perylene - based system that is probably less challenging synthetically and that also has the required properties. Again, we give citations of papers that may aid in its synthesis.

We do not claim that we offer the optimal list of polymer architectures from a synthetic point of view. We use the proposed structures as examples to emphasize that the goals of the organic synthesis should be to create polymer architectures with very strained and tight (up to VdW) intermonomer distances while at the same preventing torsions and slippages between the monomers that reduce the electronic coupling. These multiple constraints are difficult to satisfy simultaneously from a synthetic point of view, but they cannot be avoided from the point of view of achieving fast triplet exciton transport over long distances. Thus, we set these constraints as a design challenge to the organic synthesis community.

We have added the relevant new text on pages 16-18.

Reviewer: 2

Recommendation: This paper is publishable subject to minor revisions noted. Further review is not needed.

Comment 1*:*

p 4 l54: In my view the condition V_rms >= \lambda/2 is correct only if lambda is defined as the reorganization energy for exciton transfer from donor to acceptor accounting for reorganization energy of _both_ molecules, donor and acceptor (lambda = lambda_D + lambda_A) However, it appears in this paper lambda is defined to be the monomer reorganization energy only (lambda_D or lambda_A) This issue is important because V_rms in their designed molecule is 0.14 eV and lambda_D + \lambda_A = 0.27 + 0.27 = 0.54 eV, so V_rms >= \lambda/2 does not seem to be fulfilled. Please clarify.

Author's answer:

Lambda is defined in our manuscript as stated by the referee i.e., (lambda = lambda_D + lambda_A). Given that we suggest a homopolymer, i.e., all monomers are the same, for any nearest neighbor pair D=A and lambda_D = lambda_A = lambda_monomer. Therefore, (lambda = lambda_D + lambda_A= 2 lambda_monomer).

Further for D=A, the activation energy for D to A transport is U_activ=lambda/4=(2lambda_monomer)/4=lambda_monomer/2. Therefore, the condition V_rms >=U_activ becomes V_rms >= lambda_monomer/2 as used originally in the submitted manuscript. We have added all these details in the text so as not to confuse the reader. We emphasize that this is an approximate indicator of exciton delocalization that seems to work for all the polymers we have tested by MD and ab-initio computations of IPR values. Please see the new text relevant to this discussion on pages 4-5.

Comment 2*:*

p10 l 30: Are the H_j,i elements on the Liouville Equation assumed to be time-independent? If so, then some important nuclear dynamics effects are probably not captured in this equation, e.g. diagonal and off-diagonal disorder, spontaneous TE localization, back reaction from electronic to nuclear dynamics, non-adiabatic transitions etc. The approximations of the Liouville equation used should be explained (the authors do say that the IPR does not change much during MD but the MD is probably just ground state dynamics with classical force fields).

Author's answer:

The Liouvillian model captures pure dephasing due to site-energy fluctuations σ_E (diagonal disorder) and also population relaxation. We include off-diagonal disorder by using rms(H_ij) rather than H_ij in the Liouvillian. Both the site energy fluctuation σ_E and the rms(H_ij) are computed from MD simulations coupled to ab-initio computations. This phenomenological approximate model is the standard <<zeroth-order>> model for describing transitions from incoherent to coherent transport and it has been used in several contexts in the past (see references 53-55 and 57 in the main text). Importantly, it also has the advantage of approximate analytical solutions for transfer times. We used it as a screening tool to probe whether a proposed bridge structure is a good candidate for coherent triplet exciton transport

and to compute the inter-bridge transport time from the analytical expressions and as a function of polymer length. If a structure fails the test of coherent transport using this model, it will certainly fail the test of a much more sophisticated simulation as described by the referee. If a structure passes the test and it can be synthesized and a donor and an acceptor are chosen, then it could be tested by the ab-initio methods.

In summary, the model does not fully capture all possible effects such as back-reactions and non-adiabatic transitions. These effects can only be determined by doing very expensive non-adiabatic dynamics coupled with ab-initio MD for excited states. But such simulations cannot be regularly done on any proposed structure consisting of long polymers. The Liouville model using computationally derived parameters serves the purpose of an initial reliable test for coherent fast transport in a proposed structure.

We now describe the limitations and purpose of the model in pages 10-13.

Comment 3*:*
p 11 l 10: The authors should explain a bit better the physical meaning of \tau_trap and \tau_br instead of referring to Ref 54.

Author's answer:
We have added text describing the model and the time scales on pages 11-13.

Comment 4*:*
Scheme 1: the bridge molecules proposed look rather strained - is there any evidence that these molecules actually exist / could be synthesized?

Author's answer:
We have addressed this comment in response to the comments of the other referee and also modified the conclusion on page 17.

Comment 5*:*
p 15 l17: 1/k_br = 10 fs Could the authors show how this value depends on the bridge length? How much faster is TE transport in this wire compared to the one measured experimentally by Vura-Weis et al?

Author's answer:
Due to the journal's limitations, we could not add an additional figure of this distance dependence in the main text, but we added one in the SI and added a discussion in the main text (page 12). We also compared to the Vura-Weis paper, but it should be noted that the Vura-Weiss paper does not directly measure the intra-bridge transfer time, it only deduces it from a phenomenological rate model. The derived time in the Vura-Weis paper is much

slower than 10s of fsecs (it is of the order of 100 psecs). We have added a comment on page 12.

<u>Comment 6</u>*:*
p 13 l 55: TD-DFT (ωB97/def2-SVP), usually one would expect a basis set of at least double zeta quality - were the results for SVP basis set checked against larger basis sets?

<u>Author's answer:</u>
In response to the referee's comments, we performed the higher-level calculations on smaller systems, and we found that the lowest 20 triplet excited-state energies computed with def2-SVP deviate only 2% at maximum with respect to the triplet excited states that are computed with def2-TZVP. In addition, the ordering of the triplet excited states does not change. Therefore, we have no accuracy issues. These new computations are now mentioned in SI section 4.

<u>Comment 7</u>*:*
p 4 l 44 One important parameter is also the thermal fluctuations of V (\sigma_V), which should be small in the coherent/delocalized regime.

<u>Author's answer:</u>
We thank the referee for point this out. Indeed, we find that the fluctuations in the coupling are small with respect to the coupling average for the proposed systems (maximum 30% of the average). We added text related to this point on pages 5 and 17.

We hope that our explanations and changes to the manuscript have addressed the comments of the referees.

All other comments from the editorial office have been addressed.

Sincerely,

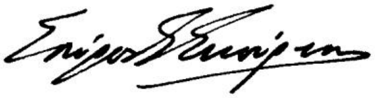

Spiros S. Skourtis

Associate Professor
